# Environmental and demographic determinants of *Aedes albopictus* seasonal activity in southern France: A modeling study

**DOI:** 10.1371/journal.pcbi.1014799

**Published:** 2026-09-10

**Authors:** Paul Taconet, Andrea Radici, Guillaume Lacour, Antoine Mignotte, Pachka Hammami, Didier Fontenille

**Affiliations:** 1 MIVEGEC (Université de Montpellier, IRD, CNRS), Montpellier, France; 2 TETIS (INRAE, AgroParisTech, CIRAD, CNRS, Université de Montpellier), Montpellier, France; 3 Altopictus, Pérols, France; 4 ASTRE (CIRAD, INRAE), Montferrier-sur-Lez, France; Department of Infectious Diseases and Hospital Epidemiology, SWITZERLAND

## Abstract

The presence and the activity of *Aedes albopictus* are a growing concern for nuisance and public health in Europe. Despite an increasing number of entomological and modelling studies, our incomplete understanding of mosquito population response to weather drivers in natural conditions restricts the development of sound vector management policies. Here, we aim to explore the role of weather conditions on *Ae. albopictus* presence and abundance in four sites in southwestern France. We rely on ovitrap longitudinal records collected on a 1–2 weeks basis and weather time series over 2023 and 2024 to model oviposition activity. Our analysis combines a mechanistic model from literature and a machine learning model fitted on cross-correlated lagged weather predictors. Temperature showed a strong association with the presence of Ae. albopictus, and captured a substantial part of the interannual variation in oviposition across sites, particularly in spring and autumn. Warm springs and autumns extend the periods in which *Ae. albopictus* life-history traits (fertility, development, survival) approach their thermal optima. In summer, a more prominent role of rain and humidity emerges among secondary drivers of oviposition intensity. Both models satisfactorily reproduce the observed oviposition dynamics, correctly predicting the onset and the end of the activity – periods that existing models have often inadequately captured. This work will also contribute to conceiving and developing operational weather-driven forecasting tools for *Ae. albopictus* activity to support vector control operations in different biogeographical contexts.

## Author summary

Weather conditions are known to strongly influence Asian tiger mosquito populations dynamics, but their effects remain poorly quantified in southern France, particularly at spatial and temporal scales relevant for vector control. In this study, we monitored mosquito oviposition in four sites of southern France between 2023 and 2024 to understand the role of different weather parameters in shaping seasonal trends. The modelling approaches we used were able to reproduce the observed trends. Temperature appears to play a major role in shaping the presence and abundance of laid eggs, especially in spring and autumn. As global warming increases temperatures in such seasons, we suggest that length of the mosquito’s activity period is likely to increase in the future. Rainfall played as a secondary driver, limiting laid eggs abundance in hot dry periods. We believe this study will help to the development of operational tools for mosquito control operations.

## Introduction

The Asian tiger mosquito *Aedes albopictus* is an arthropod native to tropical rainforest of Southeast Asia [1]. In the last century, facilitated by globalization of shipping, it invaded both tropical and temperate areas worldwide [2]. In mainland France, its first establishment occurred in 2004 in the southeastern regions [3]. Its biting nuisance and its competence to transmit arbovirus, such as dengue and chikungunya, have raised widespread concerns about its control. In mainland France, the first autochthonous transmission of these arboviruses occurred in 2010 in Nice (dengue) and Fréjus (chikungunya; [4]. Consistently with the intensification of outbreaks in tropical areas, the number of autochthonous cases of *Aedes*-transmitted arboviral disease have gradually increased in Southern Europe, reaching 833 cases in mainland France in 2025 and 357 cases in Italy in 2024 [5,6].

The need to both anticipate and mitigate biting nuisance and vector-borne disease risk has prompted vector control operators, public health officials, and citizens to try to interpret the seasonal dynamics of *Ae. albopictus* under the light of possible drivers. These stakeholders adapt their behavior following decision rules based on a range of models, from simple approaches, as calendar dates [4], to complex model ensembles [7]. Ultimately, most models assume – either implicitly or explicitly – that the seasonal dynamics of *Ae. albopictus* depend on weather conditions. Eggs need water, usually provided by precipitations, to hatch and develop into aquatic stages [1]. Temperature affects *Ae. albopictus* development, survival and fertility [8]. Humidity is also considered as important for adult survival, and wind patterns plays a minor role on its dispersion [9]. Beside these broad principles, there are evidences that *Ae. albopictus* populations exhibit signatures of local adaptation to novel selective pressures, reflecting evolutionary processes that facilitate the establishment in diverse environments. Populations in western Madagascar are adapted to endure long dry periods [10]; in temperate areas, they survive to winter thanks to photoperiodically-induced egg diapause, which evolved rapidly during invasion of poleward areas [11,12], with eggs being resistant to colder temperature compared to their tropical counterparts [13]. These adaptive capabilities challenge our possibility to extrapolate current knowledge about *Ae. albopictus* into newly colonized areas, making it more difficult to anticipate adapted measures for the management, surveillance, and control of this species. Despite growing research effort to clarify the role of weather determinants of *Ae. albopictus* activity [14–19], local authorities, public health services and mosquito control agencies often lack of solid scientific evidence to support their policies. These are ultimately jeopardized by climate changes, expected to affect the seasonality of this vector [20].

Our study aims to fill this gap and enable better anticipation of the nuisance and health risk associated with *Ae. albopictus*. We focus on four different ovitrap longitudinal records collected on a 1–2 weeks basis and weather time series over 2023 and 2024 in Occitanie and Nouvelle-Aquitaine, southern France, which have been colonized after 2010. We model oviposition dynamics using both machine learning-based and mechanistic approaches, and compare their predictions with ovitrap observations. For these sites, we explore the importance of weather and weather-driven demographical determinants of mosquito trends. Therefore, we statistically infer the presence of an interannual trend in terms of ovipositing activity. Finally, we conclude on some remarks about the possibility of forecasting ovipositing activity and its implications for optimizing mosquito control operations in the context of the climate change.

## Results

### Analysis of oviposition and weather time series

**Fig 1:**
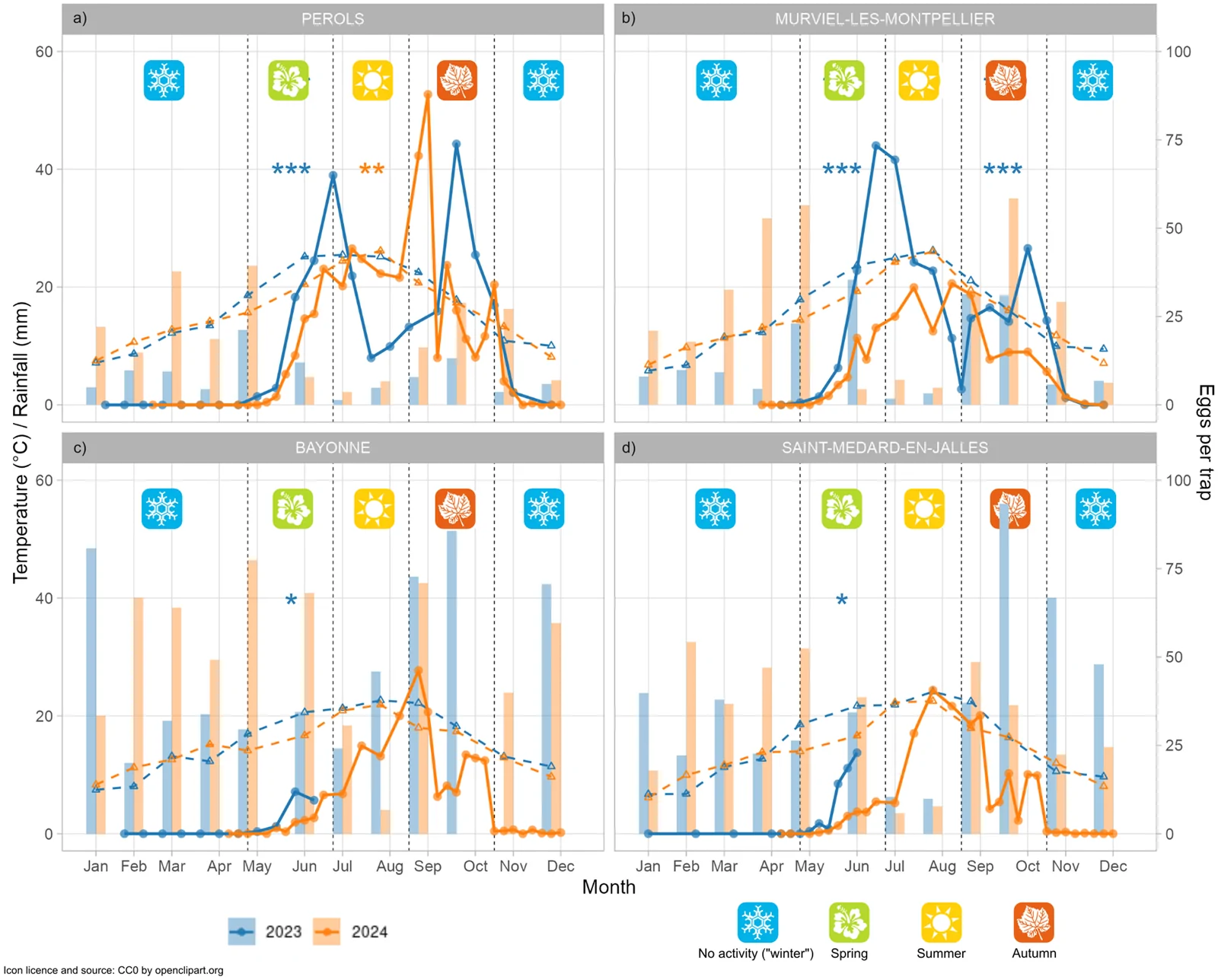
Ovitrap data (solid line; mean value for each record) in 2023 - 2024 across the sites, together with average daily temperature (dashed line; averaged with a monthly moving window) and monthly rainfall (bars). The stars indicate the p-value (*: < 0.05, ***: < 0.01, ***: < 0.001) according to the one-to-one Wilcoxon test between ovitrap data (blue: 2023 is significantly greater, orange: 2024 is significantly greater).

Compared to 2023, the oviposition onset of 2024 occurred later in the season for each site except Bayonne (Fig 1, Table D in S1 Appendix). For both Pérols and Murviel-les-Montpellier, the last positive ovitrap was found later in the year in 2023 compared to 2024. Despite the variability of ovitraps records, the Wilcoxon test revealed the presence of a significant difference in observed oviposition abundance between seasons. In Pérols, spring oviposition was significantly higher in 2023 than in 2024, while summer oviposition was significantly higher in 2024; autumn oviposition was higher in 2023, but not significantly (Fig 1a). In Murviel-les-Montpellier, oviposition was always higher in 2023, but this difference was not significant in summer (Fig 1b). In Saint-Médard-en-Jalle and Bayonne, spring oviposition was higher in 2023 than in 2024 (Fig 1c,1d). Every season in every site was warmer in 2023 compared to 2024 (with the exception of summer in Bayonne and Saint-Médard-en-Jalle, in which temperatures remained roughly stable). Except for Pérols, where rainfall was higher in every season in 2024 compared to 2023, there was no clear interannual trend in precipitation (Tab. SI4 in S1 Appendix).

### Models performance assessment

**Fig 2:**
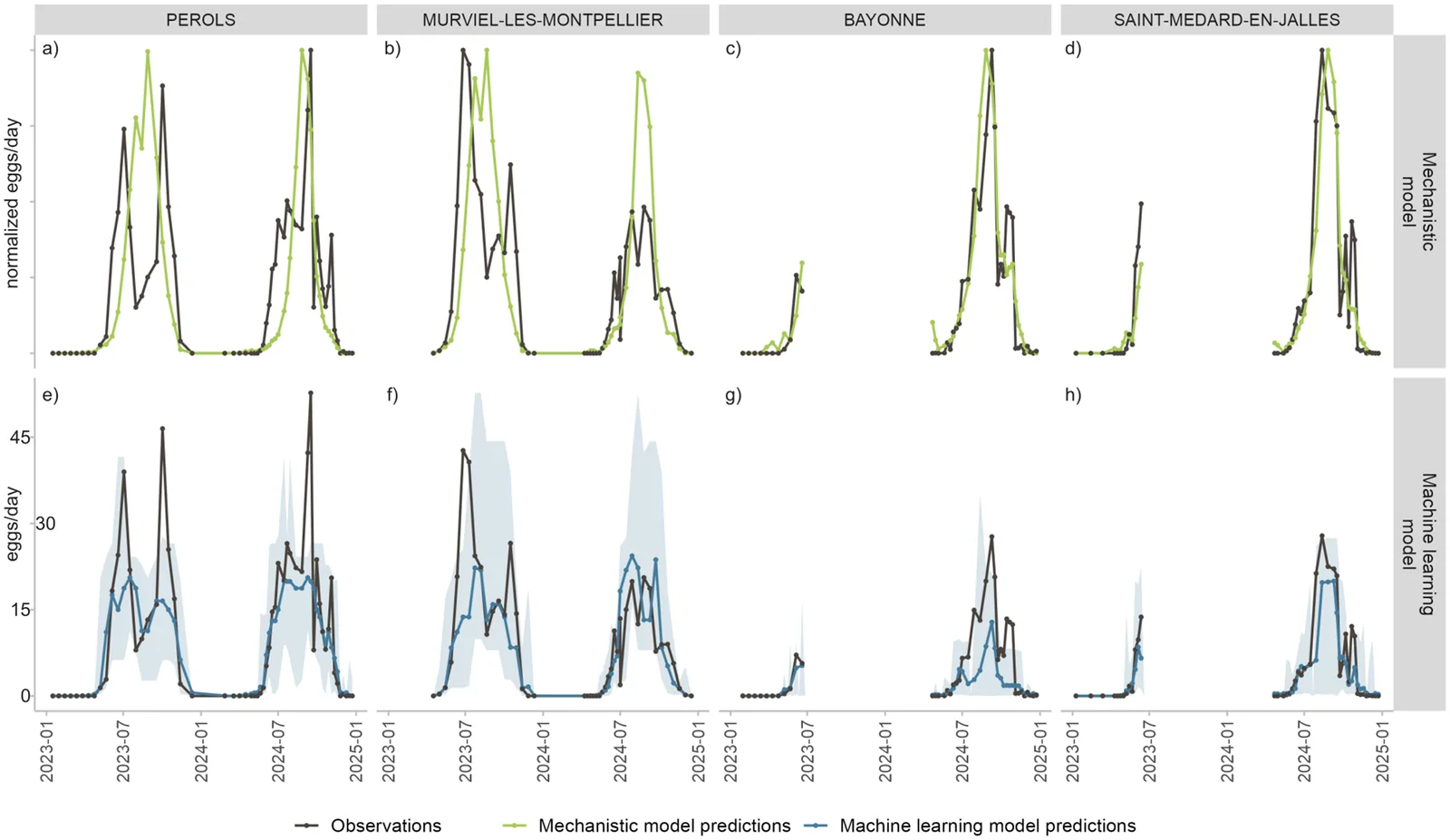
Observed versus simulated oviposition across sites. First row: normalized observations compared with predictions from the mechanistic model. Second row: absolute observations compared with predictions from the machine-learning model. The shaded ribbon represents the uncertainty of the machine-learning predictions, corresponding to the 5th–95th percentile interval derived from the quantile regression forest.

Both the presented ML model and the mechanistic model from the literature [17] globally captured the seasonal trend (Fig 2). The beginning and the end of the seasons were globally better captured by ML models, especially in those locations where the whole time series was available (Pérols, Murviel; Fig 2e, 2f). In the same locations, ML models were particularly good at capturing the inter-seasonal trends (peaks and downs). The predictions of the models were always significantly close to the observations (Table 1). Paired bootstrap comparisons indicated that differences in correlation between the ML and the mechanistic models were not statistically significant across all sites except Pérols, where the ML model achieved significantly higher correlation than the mechanistic model (Table F in S1 Appendix).

**Table 1:** Performances of the models. All the correlation values are significative at a p-value < 0.001.

Site	Mechanistic model		‍Machine learning model		
	All time series Spearman r	Abundance time series Spearman r	Presence model	Abundance model	
			Leave-one-site-out AUC	Leave-one-site-out Spearman r	Leave-one-site-out MAE
Pérols	0.87	0.70	‍0.96	0.83	9.63
Murviel-les-Montpellier	0.89	0.84	‍1	0.80	8.45
Bayonne	0.88	0.86	‍0.97	0.82	6.88
Saint-Médard-en-Jalle	0.92	0.90	‍0.95	0.90	3.95

### Lagged associations between weather and oviposition dynamics

We computed cross-correlation maps (CCMs [21]) to investigate the temporal relationships between weather conditions and mosquito activity, which revealed distinct temporal patterns for eggs presence and abundance across the study sites (Figs B and C in S1 Appendix). Temperature-related variables – average, minimum, and maximum temperature – consistently emerged as the most correlated predictors for both presence and abundance. For presence, the strongest associations are generally observed at 1–8 weeks lags, while for abundance, they are observed at shorter time lags (1–4 weeks). Relative humidity and rainfall-related variables displayed weaker and more heterogeneous correlations, with peak correlations generally occurring at longer time lags (up to 12 weeks). Wind-related variables generally exhibited the weakest associations with mosquito dynamics, with few exceptions. The temporal structure of associations displayed a notable degree of consistency across sites, especially for temperature.

We trained two types of Machine Learning (ML) models, both based on Random Forest (RF) techniques [22]. For the first weather-based presence/absence model we selected (i) average temperature between the 1^st^ and 9^th^ week (preceding oviposition; TM_0_8), and (ii) relative humidity from the 6^th^ to the 12^th^ week (UM_5_11). For the abundance model, the retained predictors included (i) average temperature from the 1^st^ to the 5^th^ week (TM_0_4), (ii) relative humidity from the 1^st^ to the 12^th^ week (UM_0_11), and (iii) cumulative rainfall from the 2^nd^ to the 6^th^ week (RR_1_5).

### Analysis of environmental determinants

#### Pooled-site effects of weather variables on oviposition activity.

**Fig 3:**
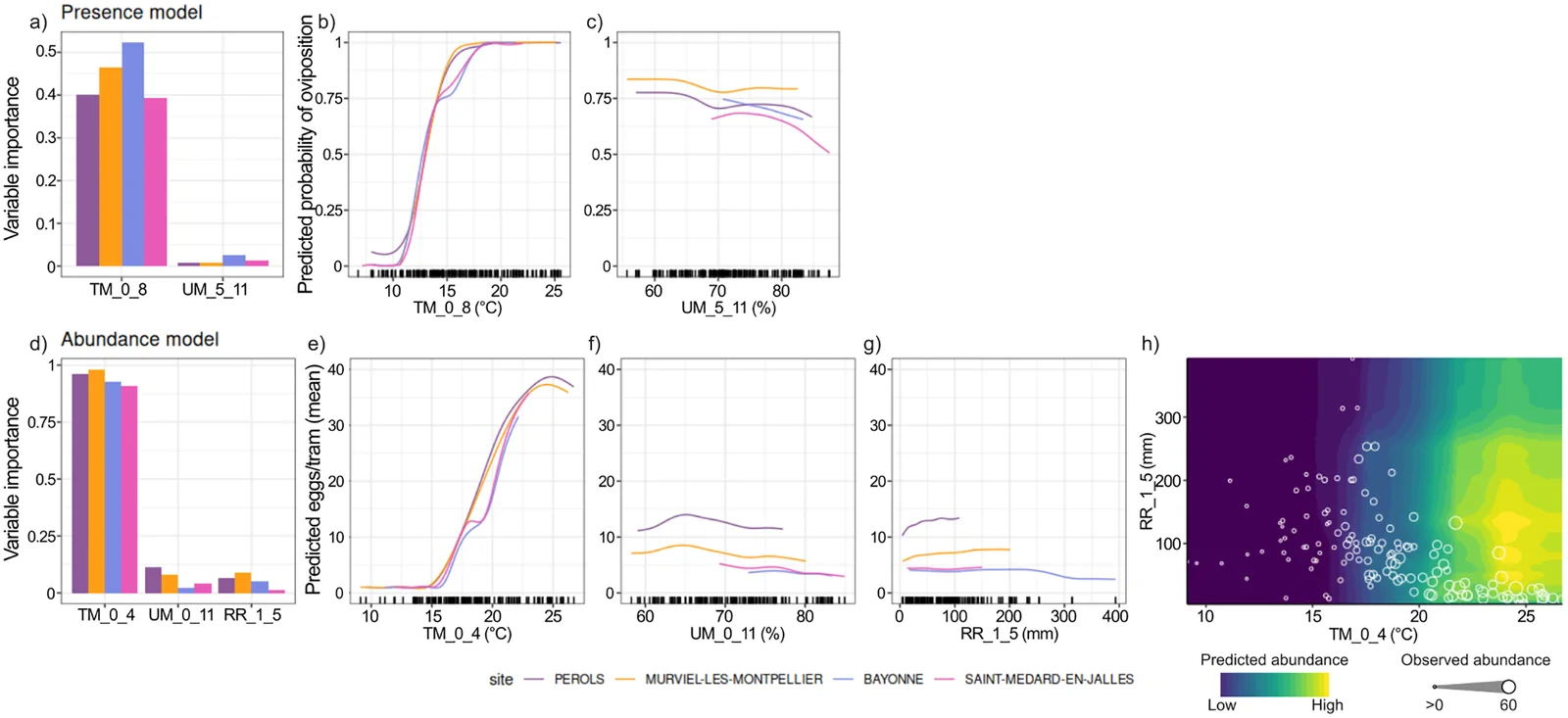
VIPs and PDPs for the mosquito presence (top) and abundance (bottom) models. Bar plots (a,d; left) show variable importance, indicating the relative contribution of each environmental predictor to the RF model. Line plots (b, c, e, f, g; right) display partial dependance, representing the marginal effect of each predictor on the response variable while averaging over the influence of other variables. The bivariate PDP in the bottom panel (h) illustrates the combined effect of temperature (TM_0_4) and rainfall (RR_1_5). For the presence model, the response variable is the probability of oviposition occurrence; for the abundance model, it is the predicted number of eggs per trap (restricted to presence-only data). These plots are based on models trained on pooled data across all sites.

Variable importance plots (VIPs) and partial dependence plots (PDPs) highlighted the dominant role of temperature in predicting both mosquito presence and abundance (Fig 3). For the presence model, average temperature between the 1^st^ and 9^th^ weeks (before oviposition; TM_0_8) was by far the most important predictor across all sites, while relative humidity during weeks 6–12 (UM_5_11) had only a minor impact (Fig 3a). The PDPs showed a sharp increase in the probability of mosquito presence as TM_0_8 rises from approximately 11°C to 18 °C, with a flection point around 13 °C, after which the probability saturated (Fig 3b). Relative humidity exhibited a weaker and more complex relationship – generally negative – with presence probability (Fig 3c).

In the abundance model, average temperature weeks 1–5 (TM_0_4) was the most influential predictor, followed by relative humidity (UM_0_11) and cumulative rainfall (RR_1_5; Fig 3d). The PDPs revealed a strong, linear increase in predicted egg abundance with TM_0_4 ranging from 15 °C to 24 °C, after which the abundance saturated and then decreased (Fig 3e). In contrast, higher relative humidity was generally associated with a decrease in predicted abundance (Fig 3f). The effects of rainfall differed across sites, showing a slightly positive effect on oviposition intensity in Pérols and Murviel-lès-Montpellier, while appearing negligible in Bayonne and Saint-Médard-en-Jalles (Fig 3g). The pooled bivariate PDP for TM_0_4 and RR_1_5 highlighted an optimal range of conditions for oviposition activity, with maximum predicted egg abundance occurring near 25 °C average temperature of the previous 5 weeks (TM_0_4) and approximately 150 mm cumulative rainfall over the 2^nd^ to 5^th^ weeks before deposition (RR_1_5; Fig 3h).

#### Local spatio-temporal effects of weather variables on oviposition activity.

**Fig 4:**
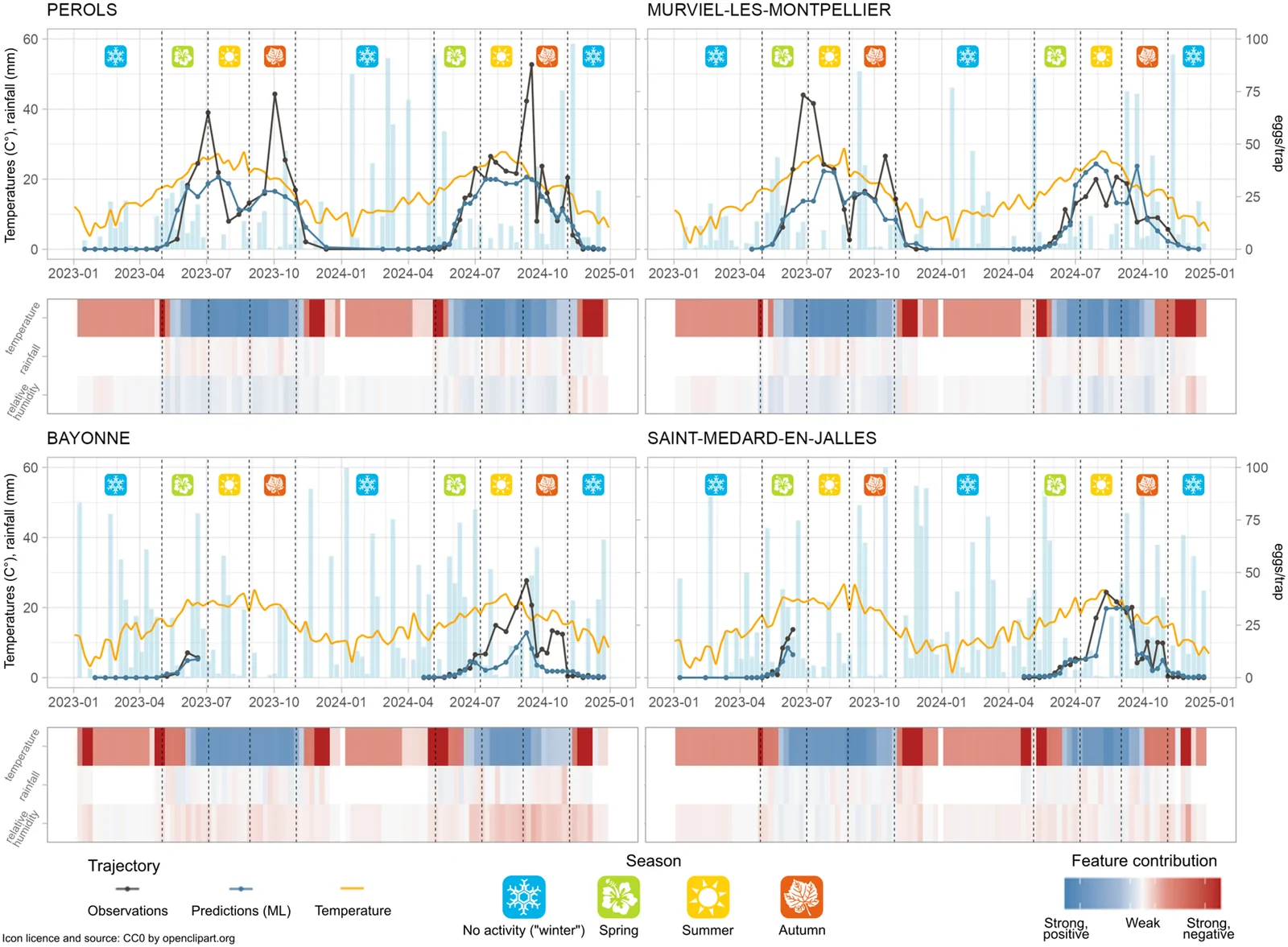
Local contribution of environmental predictors to predicted mosquito abundance using LIME. Each colored tile represents the contribution of a given environmental variable to the model’s predicted egg presence/ and abundance for a specific site and time point (weekly resolution, 2023–2024). Colors indicate whether the variable contributed positively (blue) or negatively (red) to the prediction, with color intensity proportional to the magnitude of the effect.

Across all sites and weeks, temperature consistently emerged as the most influential predictor driver, with strong positive contributions aligning closely with observed peaks in egg abundance, particularly during the warmer months (Figs 4, and S5-S8). Rainfall and humidity, by contrast, showed more variable and context-dependent effects. Notably, in Pérols and Murviel-lès-Montpellier, distinct declines in predicted abundance were observed during summer (Fig 4a,4b). In this season, temperature remained positively associated with oviposition intensity, whereas rainfall made a negative contribution to the model predictions.

### Analysis of weather-driven mosquito demographic determinants

**Fig 5:**
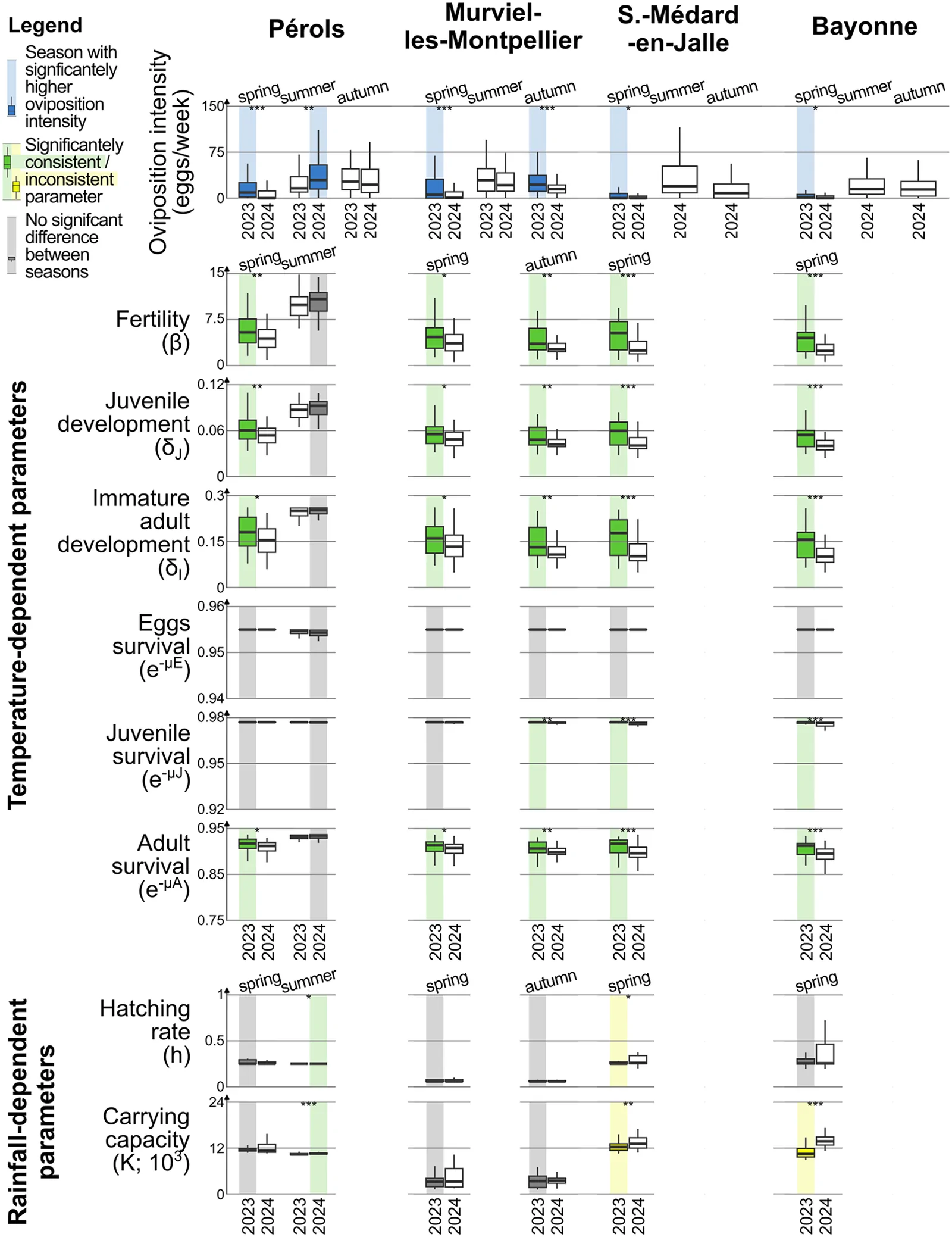
Consistency analysis per site, year and season between oviposition intensity (boxplots in the first row) against mechanistic weather-driven parameters (boxplots from the second row). For each site and season, the boxplots corresponding to the year of highest oviposition are colored in blue. Then, the boxplots of those combinations of site and season in which the parameters varied consistently(/inconsistently) with oviposition are colored in green(/yellow). Gray boxplots mean lack of significance. The stars indicate the p-value (*: < 0.05, ***: < 0.01, ***: < 0.001) according to the one-to-one Wilcoxon test.

The Wilcoxon test reveled a significance difference in oviposition intensity different between 2023 and 2024 for selected seasons and sites. Using the same statistical framework, we analyzed if weather-dependent demographic rates of the mechanistic model varied consistently with the ovitrap observations for these sites and seasons. With the exclusion of egg survival, temperature-related parameters were globally consistent with ovitrap observations (Fig 5). Fertility (β), juvenile development ($\delta_{J}$), immature adult development ($\delta_{I}$) and adult survival ($e^{-\mu_{A}}$) were consistent 5 times out of 6, juvenile survival ($e^{-\mu_{J}}$) 3 out of 6. In all the other cases, the trend was neither significant nor inconsistent. By contrast, observed oviposition was consistent with rainfall-dependent demographic traits – hatching (h) and carrying capacity (K) – only in summer in Pérols. In all other cases, they were either not significant or inconsistent (i.e., they significantly varied in the opposite way as the observations; further details in Fig I in S1 Appendix).

## Discussion

### Oviposition activity is strongly associated with average temperatures of the previous weeks in Southern France

In the study area, a limited number of weather drivers, affecting different mosquito life-history traits, explain interannual trends of ovipositing activity – a widespread proxy used to monitor the activity of adult females [23]. Sustained temperature conditions over approximately the preceding two months were the main determinant of egg presence, while temperature over roughly the preceding month showed the strongest association with egg abundance. The relatively long temperature lag identified for presence suggests that mosquito activity is driven by sustained thermal suitability over several weeks, consistent with the progressive activation of populations following diapause. The importance of considering lagged temperatures into the description of oviposition dynamics in southern Europe is confirmed by recent research – despite disagreeing with these studies on the amplitude of the lag. For instance [24], found that average temperature of the previous 5 weeks is the most correlated driver to observed oviposition intensity in Palermo, Italy, while [7] found that average temperature of the third-to-last week is the most important determinant of oviposition intensity over the area covered by the VectAbundance survey in southeastern Europe [23]. These differences may reflect either local population dynamics or the results of different modelling choices. Concerning diapause end, it is interesting to notice that our results allow to correctly capture and satisfactorily explain the onset of mosquito activity also neglecting the photoperiod, the main driver of egg diapause (i.e., the population overwintering through the production of eggs that will hatch in spring; [11,25,26].

#### Warm temperatures facilitate longer and more intense activity seasons of *Ae. albopictus* in a temperate climate.

Wide research focuses on temperature as the main driver of the ecology of ectotherm populations [8]. formalized the performance of a corpus of mosquito life-history traits as an (asymmetric) bell-shaped function of temperature [9,27]. The optimal thermal response of *Ae. albopictus* on several key biological traits varies between 24.2 °C (survival of aquatic stages) to 32.6 °C (adult development rate; [28]). For temperatures lower than the optimal ones, as it regularly occurs in temperate countries, a temperature increase corresponds to an increment of mosquito fitness [28]. Our analysis of demographic determinants in the mechanistic model corroborates this idea; temperature-dependent traits (i.e., survival, development or fertility rates) varied consistently with oviposition intensity across all sites during cool seasons (spring and autumn) characterized by suboptimal temperatures.

It is worth noticing that climate change increases the likelihood of warmer springs and autumns; in a temperate climate such as that of southern France, the increase of temperatures would lead to the extension of the activity season of the mosquito, as other studies pointed out [29,30]. In addition, warm temperatures decrease the extrinsic incubation time of arboviruses, which develop and are transmitted more easily, therefore increasing the period at risk of transmission of arboviruses [20,31,32]. As a consequence of this two factors, it is the advisable that the calendar for enhanced surveillance, which currently begins in May in mainland France [4], should be adjusted accordingly.

### Limited role of rainfall and humidity in shaping mosquito activity in southern France

The analysis of environmental determinants indicates that rainfall likely plays a secondary role in shaping population abundance dynamics, while it does not affect the presence. Consistently, the analysis of environmentally-driven demographics suggests that rainfall becomes determinant only during summer. The primary effects of rainfall are to trigger egg hatching and to increase the availability of larval breeding sites, the main limiting resource to mosquito population growth [9]. Yet, the importance of rainfall in sustaining larval survival – and therefore mosquito population – in urban settings is increasingly debated [33]. Urbanization has led to an increased capacity to retain water – whether from rainfall or irrigation – due to the expansion of impervious surfaces (gutter silt traps, raised terraces, roadworks, flowers pots, rainfall collectors, etc.). In many cases, human irrigation compensates for reduced precipitation, providing persistent larval refugia that reduce mosquito populations dependence from natural water supply [34]. It is not surprising that rainfall-dependent parameters only become the limiting factors to oviposition only during the driest season in the driest locations (~ 21 mm of cumulative rainfall in June and July in Pérols, while Bayonne has ~ 110; Tab. SI4 in S1 Appendix). Moreover [35], suggested that, during autumn, females laying diapausing eggs exhibit a preference for larger containers; this behavioral shift reduces the dependance of the first generations of larvae on rainfall abundance (or scarce evaporation). Interestingly, rainfall appears to play a context-dependent role, with a weak but positive association in Mediterranean sites (Pérols, Murviel) and no detectable effect in more oceanic settings. In Mediterranean environments, where summer conditions are typically dry, rainfall events may temporarily create or replenish breeding sites and alleviate desiccation stress, thereby supporting oviposition. In contrast, in oceanic regions where moisture is more consistently available, water availability is less likely to be a limiting factor, and rainfall does not emerge as a key driver of mosquito activity. These results suggest that the influence of rainfall is modulated by the background hydrological context, acting primarily as a limiting factor in drier environments but becoming negligible where suitable conditions are already sustained.

The VIP analysis highlights a secondary role of relative humidity, which is associated with a small negative effect on both the presence and abundance of oviposition. Relative humidity has long been recognized as a challenging variable to embed into a modelling scheme due to its complex interactions with other weather variables [9,36]. For instance, high humidity is expected to enhance eggs survival at 26 °C, but the same does not hold consistently across other temperatures [37]. The effect of humidity on adult mosquitoes is even less understood: European populations of Asian tiger mosquito have been found also in regions with relative humidity levels below 35% [9]. In our study, relative humidity was retained following the data-driven variable selection procedure and after accounting for collinearity with other predictors, suggesting that it contributes additional information to the model. However, its contribution remained substantially weaker than that of temperature, and its ecological interpretation should therefore be treated with caution. In particular, the relatively narrow range of values observed in the study area (between 60 and 85%) may limit its detectable influence on mosquito dynamics. As a result, we interpret relative humidity primarily as a secondary atmospheric covariate that may capture local environmental conditions not fully represented by temperature and rainfall, rather than as a dominant driver of oviposition dynamics.

### The effects of extreme weather events on the mosquito activity in southern France

Summer temperatures may exceed the optimal range of *Ae. albopictus*. This occurred in the warmer sites of Pérols and Murviel-les-Montpellier, where the PDP reveal a slight decrease of abundance for average monthly temperatures exceeding 24 °C. While on-field evidence of this phenomenon in temperate climate is poor, other modelling studies document the negative effect of summer heatwaves on mosquito fitness [38]. As a matter of fact, it is difficult to disentangle the effect of heatwaves from that of droughts, as in temperate climate they often occur simultaneously. Notably, in Pérols and Murviel-lès-Montpellier, distinct declines in predicted abundance were observed during summer 2023, despite suboptimal but still favorable temperature conditions. In these dates, the LIME analysis provided an insight on the role of low rainfall, which makes a negative contribution to the model predictions, suggesting that low or insufficient precipitation results in limited oviposition. In contrast, in Bayonne and Saint-Médard-en-Jalles, rainfall and humidity show minimal or inconsistent influence across the season, indicating a reduced predictive value of these variables under the prevailing local oceanic conditions.

Across the available time series, we have not observed extreme rainfall events. Several studies reported that excessive rain may flush out breeding sites, potentially disrupting mosquito population dynamics [9]. However, the 2014 epidemic of chikungunya in the Montpellier area – which includes Pérols and Murviel-les-Montpellier – following an exceptional rain event (299 mm on the 29th of September) lead researchers to reconsider the effect of extreme precipitation. In such cases, heavy rainfall may also create an unprecedented number of new breeding sites, ultimately boosting the mosquito population [39,40]. While our analysis highlights that rainfall plays a secondary role in driving oviposition intensity, the PDPs indicate that cumulative rainfall exceeding 150–250 mm over the preceding five weeks may be associated with a decrease in oviposition intensity.

### Limitations of the study

Some caveats must be considered before generalizing the outcomes of this study. The results we obtained – analyzing a relatively short longitudinal dataset – need to be contextualized within a specific environment in terms of landscape, climate and social features – specifically, a temperate climate within a western European urban fabric. These highly anthropized sites are located approximately at the same latitude, close to the coasts. We believe that our results can be generalized to other contexts that share similar environmental features in terms of climate and urbanization. However, extending entomological observations both in time and space would help capturing a wider range of environmental conditions and associated mosquito responses. This would help solving site-specific discrepancies (such as underestimating extreme abundance peaks), typical of ML modelling, ultimately strengthening its operational value for vector control.

Some obstacles to the generalizability of any study on *Ae. albopictus* are its high phenotypical plasticity and adaptation capability, for example of diapause, whose rapid evolution due to the latitudinal expansion of this species requires constant surveillance. Although photoperiod has a minor role in our study, it remains the primary regulator of winter egg diapause, and its evolution would alter this species’ seasonality. Some studies suggested that year-round activity may become possible in the future due to climate change in Southern Europe [30].

We compared oviposition abundance, mean temperature and cumulative rainfall between the two years using the Wilcoxon test, applied within each season and site. Each sample consists of temporally autocorrelated data, collected over consecutive weeks; therefore, they do not fully satisfy the test’s independence assumption. This tends to inflate the apparent significance of the differences, so the interannual comparisons should be interpreted with caution.

Finally, the identification of relevant lag structures was based on bivariate cross-correlation analyses, which are sensitive to shared seasonality and correlations among predictors. As a result, the selected variables and lags should not be interpreted as independent causal effects, but rather as indicative of dominant temporal scales of association within a correlated climatic system. In particular, variables with weaker seasonal signals, such as rainfall, may be underrepresented despite potentially important short-term effects.

### Directions for future research: Weather-driven models as decision support systems in mosquito management

Our models accurately predicted the onset of mosquito activity after diapause, a hard benchmark for many entomological models, as well as variations of abundances during the mosquito activity season. These results reinforce the complementary of data-driven and mechanistic tools to analyze vector dynamics – as previously noted by [41] in La Réunion – but also their potential to support operational interventions. The outcome of these models could assist vector control operators in prioritizing periods or geographical areas for vector control interventions, or enable health authorities to activate targeted surveillance activities. For instance, the early prediction of the spring onset of mosquito activity would allow for the planning of timing anti-larval interventions. Similarly, health services and mosquito control operators may be mobilized as average temperatures exceed ~18°C over a month, at which we obtained the steepest abundance increase, which has consequences over arbovirus transmission. The integration of such modelling outcomes into the routines of mosquito control operators, health services, and local authorities would allow quick actions to abate the health risk.

While examples of similar operational tools exist – such as the ARBOCARTO framework [42], which is already used in some regions of France and abroad, or the early warning system described by [43] and used in the Caribbean – there remains substantial room to improve their societal uptake. The development of ARBOCARTO highlights that creating effective tools to predict mosquito activity requires strong coordination among a wide range of stakeholders, which is not limited to the collaboration between field entomologists and ecological modelers. Beyond the scientific community, such an effort calls for collaboration between public health authorities, vector-control operators, local political officials, urban management services, and representatives of civil society. Only through this integrative, multi-actor approach can surveillance tools be effectively translated into actionable strategies for public health and vector control. Lastly, to enhance the effectiveness of these models and tools, they should be designed to forecast mosquito activity (in the future) – and not only predict it (in the present) - and to capture short-term fluctuations and peaks that are essential for guiding timely vector control actions.

## Materials and methods

### Entomological longitudinal analysis

**Fig 6:**
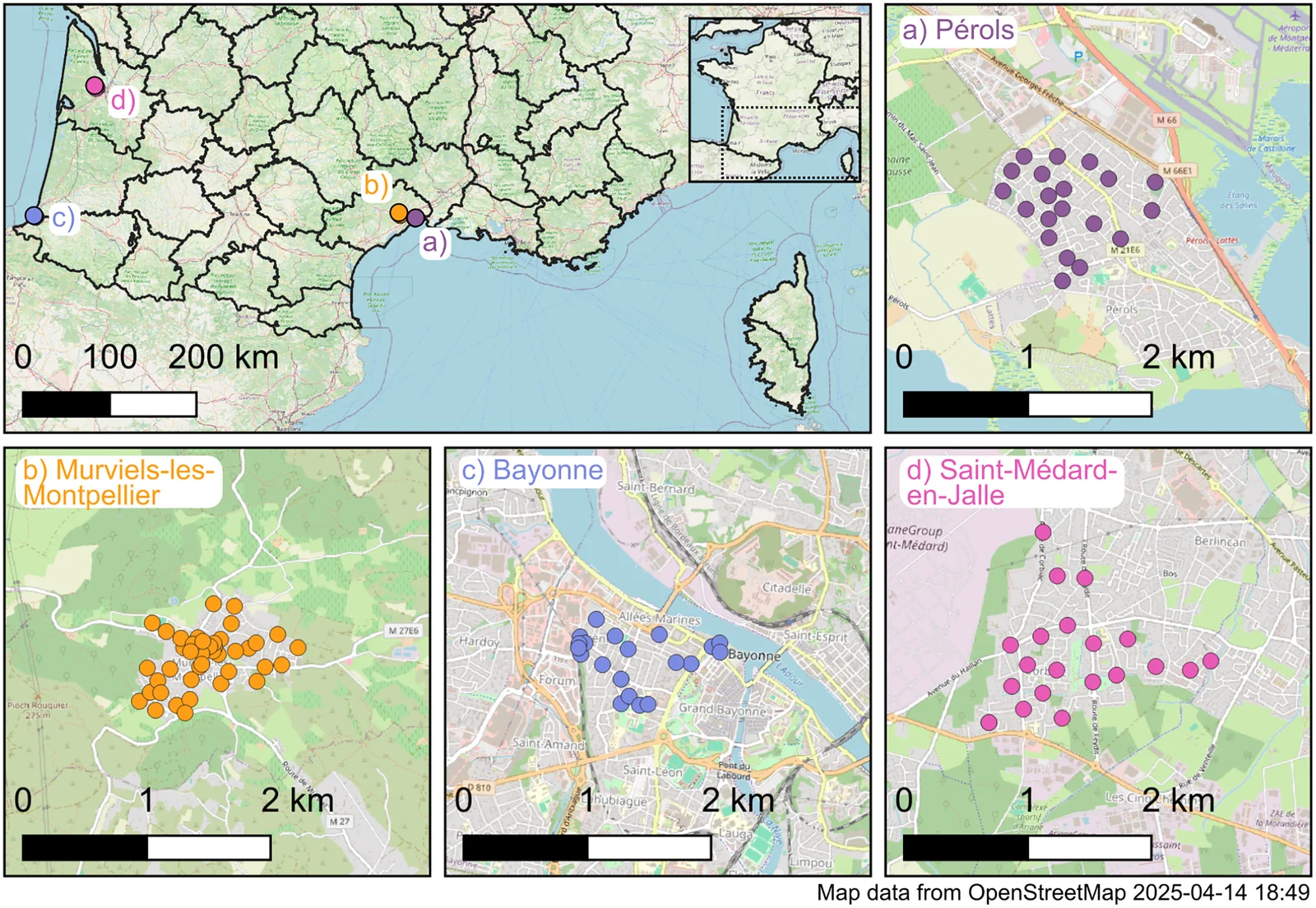
Spatial setup of the Altopictus ovitrap surveillance network in southwestern France: a) Pérols, b) Murviel-les-Montpellier, c) Bayonne, d) Saint-Médard-en-Jalle Base map from OpenStreetMap and OpenStreetMap Foundation (https://www.openstreetmap.org/copyright).

Our analysis based on entomological surveillance observations provided by the Altopictus vector-control agency ovitrap collection in four different locations in mainland France, *i.e.,* Pérols and Murviel-les-Montpellier (Occitanie), Bayonne and Saint-Médard-en-Jalle (Nouvelle-Aquitaine; Fig 6). According to the Corine Land Cover classification, the monitored areas correspond to discontinuous urban fabric, whereas some ovitraps in Bayonne were located within continuous urban fabric [44]. Ovitraps consist of artificial egg-laying containers made of 3-Liter black plastic buckets filled with 2 L of Bti-treated (*Bacillus thuringiensis israelensis* biolarvicide) tap water. A floating polystyrene square (5 x 5 x 2 cm) provided a substrate for oviposition. They are used to monitor the oviposition intensity, *i.e.,* the abundance of laid eggs, a proxy of the density of locally active females. A total of 20–30 ovitraps were deployed in each site, which have been inspected every 1–2 weeks between 2023 and 2024 (Tab. SI4 in S1 Appendix). Given the biweekly sampling at some sites, onset, offset, and peak activity should be interpreted as observed rather than exact dates, with an uncertainty of up to one sampling interval. Surveillance operations were interrupted from June 2024 onwards in Bayonne and Saint-Médard-en-Jalle. Records have been aggregated using the mean value for each inspection date and site.

To assess the impacts of different weather conditions over oviposition activity, we first analyzed the ovitrap series site by site. Each time series was divided into three seasons, *i.e.,* “spring” (May-June), “summer” (July-August) and “autumn” (September-October). For each site and season, we determined the presence of an interannual trend via the two-tailed two-samples Wilcoxon test by comparing the statistical distribution of the ovitrap records of the two years of collection.

### Input data

Both mechanistic and statistic models are driven by weather inputs. We retrieved daily precipitation (mm), daily mean, maximum and minimum temperatures (°C), relative humidity (%), average and maximum wind speed (m/s) from MétéoFrance (details in Tab SI4 in S1 Appendix). We retrieved human population density raster at 30”, aggregated over a grid of 0.1°, based on the 2015 GPWv4 data for 2015 [45].

### Machine learning model

In the machine-learning framework, oviposition was defined as the response variable, measured as the mean daily egg count for each site and collection date (i.e., the cumulative number of collected eggs, divided by the length of the sampling period, averaged over all ovitraps). Two complementary modeling components were developed. The first, referred to as the presence model, aims to identify the environmental conditions associated with mosquito activity using a binary response (presence vs. absence of eggs). The second, referred to as the abundance model, aims to predict oviposition intensity as a continuous variable (mean number of eggs per trap per day), restricted to positive observations. This two-step approach was adopted to model occurrence and oviposition intensity independently using dedicated machine-learning algorithms. This allowed each component to have its own predictor selection, model optimization and ecological interpretation, while retaining the flexibility of non-parametric RF models. Similar two-step modelling strategies have been used in ecological studies of zero-inflated responses using ML [46–48].

To identify relevant temporal scales of association between weather variables and oviposition, we computed cross-correlation maps (CCMs) between the response variable and weekly averaged weather variables at lags ranging from 0 to 12 weeks prior to each trapping date. CCMs enable the visualization of lagged associations between time series and reveal, in most cases, broad peaks of high correlation spanning several consecutive weeks rather than a single optimal lag. Based on these patterns, predictors were constructed as averages over the corresponding lag windows, capturing sustained environmental conditions rather than short-term fluctuations. The strength of these associations was quantified using the distance correlation coefficient [49], which captures both linear and non-linear relationships and is therefore well suited to complex ecological systems. CCMs were computed separately for each site to explore local patterns and pooled across sites to identify generalizable relationships.

Predictor selection followed a data-driven but interpretable approach. Weather variables showing weak associations with the response (maximum lagged distance correlation < 0.1) were excluded. To limit redundancy and because our objective included the ecological interpretation of the models, multicollinearity was reduced using pairwise distance correlation (threshold r > 0.7), retaining predictors based on ecological relevance and interpretability. Correlation matrices are provided in the SI4.

The selected predictors were then used to train data-driven predictive models based on Random Forests [45]. Classification forests were used for the presence component, while quantile regression forests (Meinshausen, 2006) were used for the abundance component, allowing the estimation of conditional prediction intervals for positive egg abundance. Model performance and generalization were assessed using a spatial leave-one-site-out cross-validation scheme, in which models were iteratively trained on three sites and evaluated on the fourth. This approach reduces overfitting and provides a robust assessment of predictive performance on unseen locations [46], while generating fully out-of-sample predictions.

Uncertainty was explicitly quantified for both modeling components. For the presence model, uncertainty was represented by the predicted probability of occurrence. For the abundance model, quantile regression forests were used to derive conditional prediction intervals (5^th^, 50^th^, and 95th percentiles). The two components were then combined into a unified prediction using a threshold-free formulation of expected abundance, defined as the product of the predicted probability of presence and the conditional abundance. To further account for uncertainty propagation, a simulation-based approach was implemented by combining Bernoulli realizations from the occurrence model with conditional abundance predictions.

### Mechanistic model

As a mechanistic model, we used the model by [17], currently used and validated in temperate areas in Europe [32,50], which describes the dynamics of the mosquito population (mosquito/ha) via ordinary differential equations into five stages (eggs E, diapausing eggs $E_{d}$, larvae and pupae as juveniles J, unfed immature female adults I, mature female adults A). These equations specify mortality, development and fertility rates, which rely on environmental (photoperiod P and human density H) and weather (rainfall R and temperature T) drivers.


$$\begin{array}{c} \left\{ \begin{array}{c} @c\dot{E}=\beta (T)\left(\text{1}-\omega (P)\right)A-\left(h{(R,H)\delta }_{E}+\mu_{E}(T)\right)E\\ \dot{J}=h{(R,H)(\delta }_{E}E+\sigma (T,P)\gamma (T)E_{d})-{(\delta }_{J}(T)+\mu_{J}(T)+\frac{J}{K(R,H)})J\\ \dot{I}={\frac{\text{1}}{\text{2}}\delta }_{J}(T)J-\left(\delta_{I}(T)+\mu_{A}(T)\right)I\\ \dot{A}=\delta_{I}(T)I-\mu_{A}(T)A\\ \dot{E_{d}}=\beta (T)\omega (P)A-h(R,H)\sigma (T,P)E_{d} \end{array}\right. \end{array}$$

Where β represent the fertility rate, $\delta_{E}$, $\delta_{J}$ and $\delta_{I}$ the development rate of eggs, juveniles and adults, $\mu_{E}$, $\mu_{J}$, $\mu_{A}$, their mortality, ω the fraction of diapausing laid eggs, h the hatching rate, σ the fraction of diapausing eggs ready to hatch, γ the probability of winter survival, K the carrying capacity of juveniles (Table A in S1 Appendix for details on weather dependencies). This model allowed to estimate the observed oviposition abundance via the indicator ${LE}_{it}$ of eggs laid per day per hectare in site at time in site i at time t:


$$\begin{array}{c} {LE}_{it}={\tilde{\beta }}_{it}(T)A_{it} \end{array}$$

Where $A_{it}$ is the simulated density of adult female mosquitoes, and ${\tilde{\beta }}_{it}$ is the temperature-dependent fertility rate. In order to compare our simulations with observed ovitrap data, we averaged ${LE}_{it}$ with a moving window of two weeks and we normalized it with respect to the highest value.

### Model evaluation

For the ML model, model performance was evaluated under a leave-one-site-out cross-validation framework to assess generalization to unseen locations. Given the two-part structure of the approach, presence and abundance components were evaluated separately. The presence model was assessed using the area under the receiver operating characteristic curve (AUC), which measures the ability to discriminate between presence and absence. The abundance model, restricted to positive observations (i.e., time steps with observed egg counts > 0), was evaluated using both Spearman correlation and mean absolute error (MAE). Spearman correlation quantifies the ability of the model to reproduce temporal dynamics, while MAE provides a measure of the accuracy of predicted magnitudes.

For the mechanistic model, evaluation was based on Spearman correlation only, as its outputs are not directly comparable in magnitude to observed egg counts, preventing the use of absolute error-based metrics such as MAE.

To facilitate comparison with the ML approach, two complementary evaluations were conducted: one using the full time series, and one restricted to time steps with observed presence, allowing a more direct comparison with the abundance component of the ML model. The statistical significance of each correlation was evaluated using a two-sided Student’s t-test.

To formally assess differences in performance between models, we implemented a paired bootstrap procedure. For each site, observations were resampled with replacement and correlation coefficients were recomputed for each model using the same resampled data. The distribution of the difference in correlation coefficients between models was then used to derive confidence intervals and assess whether observed differences were statistically meaningful. This comparison was performed on the abundance component only (i.e., using positive observations), where correlation-based evaluation is most relevant and directly comparable across models.

### Analysis of weather determinants of oviposition

To investigate the role of weather drivers in shaping oviposition dynamics, we interpreted the trained ML models using Variable Importance Plots and Partial Dependence Plots, two widely used Interpretable Machine Learning tools (Molnar 20219). VIPs measure each variable’s contribution to the model performance, while PDPs visualize its average effect on predictions, accounting for interactions with other predictors. These tools enable to highlight which environmental variables mostly influence mosquito presence or abundance.

To complement these global interpretations with fine-scale ecological insights, we applied the Local Interpretable Model-agnostic Explanations (LIME) method [51]. LIME approximates the complex ML model locally using a simpler and interpretable surrogate (e.g., a linear model). It provides local contributions of each predictor to individual model predictions, indicating both the direction (positive = increasing, negative = decreasing) and the magnitude of influence relative to other predictors at that specific site and time point.

### Analysis of weather-driven demographic determinants of oviposition

For the sites and seasons in which the Wilcoxon test of ovitrap abundance revealed a significant difference between 2023 and 2024 (p-value < 0.05), we analyzed the statistical significance of the weather-dependent demographic rates (as in [52]) computed in the mechanistic model between those two years using the same Wilcoxon test. We considered the fertility (β), juvenile development ($\delta_{J}$), immature adult development ($\delta_{I}$), egg survival ($e^{-\mu_{E}}$), juvenile survival ($e^{-\mu_{J}}$), adult survival ($e^{-\mu_{A}}$), hatching (h), carrying capacity (K). The carrying capacity was considered as a demographic trait as its role in intraspecific competition makes it proportional to survival rate. By testing the inter-annual variability of those rates, we identified those that varied the most “consistently” with oviposition, and can be considered as determinants of mosquito activity.

We employed “inconsistent” as a synonym for “significantly sharing the opposite interannual trend as the observed oviposition” and, “consistent” as a synonym for “significantly sharing the same interannual trend as the observed oviposition”.

### Software

All analyses were conducted using open-source software. The R programming language [53] served as the primary platform. The Wilcoxon statistical analysis of time series was performed using the ‘stat’ package (version 4.4.1). Correlation analyses were performed with the ‘correlation’ package (0.8.8, [54]). RF models were fitted using the ‘caret’ (7.0-1; [55]) and ‘ranger’ (0.17.0) packages, while spatial folds for cross-validation were generated with the ‘CAST’ package (1.0.3). Model interpretability analyses relied on the ‘vip’ (0.4.1; [56]) and ‘pdp’ (0.8.2; [57]) packages to produce variable importance and partial dependence plots, respectively, and the ‘lime’ package (0.5.3; [58]) to perform the LIME analysis. Among the inputs of the mechanistic model, we retrieved the photoperiod (in hours) using the ‘suncalc’ (0.5.1), based on the latitude, longitude and date.

## Supporting information

S1 Appendix Table A – Metelmann model parameters. Fig A – Layout of the weather-driven Metelmann model describing *Ae. albopictus* dynamics, edited from [32]; **Table B** – Weather variables and specifications; **Table C** – Summary about ovitrap and weather recording per site; **Table D**– Extended summary of ovitrap and weather data per site, year and season; **Fig B** – Cross correlation maps for each site (rows) and environmental variables (columns) against ovitrap observations of presence and absence; **Fig C** – Cross correlation maps for each site (rows) and environmental variables (columns) against ovitrap observations of oviposition abundance. **Fig D** – Distance correlation coefficient between candidate weather variables (left: presence model, right; abundance model); **Table E**: Predictive performance of the models for each study site; **Table F** – Results of paired bootstrap comparisons between the mechanistic and machine-learning models for each site; **Fig E** – Local contribution of environmental predictors to predicted mosquito abundance in Pérols using LIME; **Fig F** – Local contribution of environmental predictors to predicted mosquito abundance in Murviels-les-Montpellier using LIME; **Fig G** – Local contribution of environmental predictors to predicted mosquito abundance in Bayonne using LIME; **Fig H** – Local contribution of environmental predictors to predicted mosquito abundance in Saint Médard en Jalle using LIME; **Fig I** – Consistency analysis per site, year and season between oviposition intensity (boxplots in the first row) against weather variables (boxplots from the second row). For each site and season, the boxplots corresponding to the year of highest oviposition are colored in blue. Then, the boxplots of those combinations of site and season in which the parameters varied consistently(/inconsistently) with oviposition are colored in green(/yellow). Gray boxplots mean lack of significance. The stars indicate the p-value (*: < 0.05, ***: < 0.01, ***: < 0.001) according to the one-to-one Wilcoxon test.
(DOCX)
