# Supplementary material for: Environmental and demographic determinants of Aedes albopictus seasonal activity in southern France: A modeling study
Source: PLoS Comput Biol. 2026 Sep 10;22(9):e1014799. doi: 10.1371/journal.pcbi.1014799 (PMC13588507; doi:10.1371/journal.pcbi.1014799)
Supplement: Supplementary file 1 [file pcbi.1014799.s003.pdf]

Dear Editor,

Thank you for taking the time to assess our manuscript “Temperature is the key weather determinant of *Aedes albopictus* seasonal activity in southern France”. We would also like to thank the three reviewers for their time, their positive assessment of the work, and their thoughtful feedbacks. The reviewer’s comments have helped us to further improve and sharpen the manuscript. Below we provide the point-by-point responses (in green) to the reviewer’s comments.

Sincerely,

Authors

Point by point reply to reviewers

Editor comments:

Overall, the reviewers find the study relevant, however, they raise several points that require revision.

One important concern relates to the interpretation of results, in particular the use of causal language. Reviewers stress that given the limited time span (two years) and number of sites, the analyses support associations and not causal drivers and that this should be acknowledged throughout the manuscript, with non-causal language used accordingly.

We thank the reviewers for raising this point. Indeed, we felt it appropriate to go so far as to define environmental covariates as “causal drivers,” as the associations identified in southern France are consistent with well-established findings in temperate-climate countries, particularly in Europe. Upon further reflection, we share the reviewers’ caution and have revised our paper accordingly.

Another request by the reviewers is a clearer justification and description of the statistical (ML) modelling choices. This includes the two-stage presence/absence and abundance approach, the selection of predictors and how they were then ultimately combined. For example, it is not clear, why lagged variables were averaged rather than included individually in the RF models, whether alternative modelling strategies were explored (e.g. the use of different lag windows), and how sensitive results are to these choices. Reviewers also note that the inclusion of uncertainty for the RF models would be appreciated. These aspects should be explained more clearly and better justified (or explored, if not yet done).

We thank the editor for this important comment. We have substantially revised the analyses and the manuscript to clarify the machine-learning modeling framework. In particular, we now provide a clearer rationale for the two-step presence–absence and abundance approach, the data-driven selection of predictors using cross-correlation analyses, and the construction of lagged variables as averages over identified temporal windows. We also clarify how both components are combined into a unified prediction using a updated threshold-free formulation. In addition, we have expanded the description of uncertainty quantification by incorporating probabilistic outputs for occurrence and prediction intervals for abundance. Overall, these revisions improve the transparency, consistency, and interpretability of the modeling approach.

If these general points, together with the additional comments raised by the reviewers, are adequately addressed, the manuscript can be considered for publication.

Reviewers' comments:

Reviewer #1:

Dr. Taconet and colleagues presented an interesting study on Aedes albopictus seasonal activity. Considering the increasing public health significance of Aedes albopictus as a vector of diseases such as dengue and chikungunya which translate to an increased need to control its abundances, the study is undoubtedly interesting and valuable. The study integrates established modeling approaches, mechanistic and random forests (RF) models, to analyze a two-years four-sites ovitrap data in France with corresponding weather time series. The conclusions regarding the extension of the active season due to warmer spring and autumn temperatures are significant for public health, specifically for adjusting arbovirus surveillance calendars.

The primary concern regarding the manuscript is the causal tone adopted by the authors in discussing the observed correlations, which are often presented as determinants or drivers. Despite the use of a mechanistic model, I think that their experimental design, sample size and analysis do not allow for a direct causal interpretation. I suggest authors to tune down the causal language throughout the manuscript.

We thank the reviewer for raising this point. As noted in our comment to the editor, in the earlier version of the manuscript we had deemed it appropriate to go so far as to define the environmental covariates as “causal drivers,” since the associations identified in southern France corroborate well-established literature from temperate-climate countries, particularly in Europe. Although our analysis employs two different modeling

approaches, we share the reviewer's caution and have modified the tone of the text accordingly.

The modeling efforts are built upon data from only two breeding seasons (and four sites). Temporally, this is a limited database, providing only two independent observations per site for the seasonal occurring events (start, peak, end of seasons). In my opinion, this makes it challenging to pinpoint the exact drivers of mosquito activity. The mosquito species (to the best of my knowledge) is well established in the investigate areas from several years (implying adaptation to local conditions). Therefore, understanding the variations in the start of the season requires data spanning multiple seasons. I would like authors to provide a detailed explanation within the manuscript addressing why the two-season dataset is sufficient to infer key factors, or, alternatively, they must explicitly and clearly outline these temporal limitations in the Discussion section.

The areas mentioned in the article have experienced relatively recent colonization by the tiger mosquito (last 15 years). The presence of this vector has indeed been monitored since then; however, datasets that include a (a) consistent and permanent number of ovitraps in areas that are relatively (b) homogeneous in terms of landscape (so as not to have to consider other spatial covariates) are not very abundant in southwestern France. These criteria led us to exclude other time series in the region (e.g., Toulouse or Marseille). For these reasons, and due to the dual modeling approach, we prefer to adjust the article's findings and further explore the limitations in the Discussion section.

Another suggestion is that the statistical model section of the Material and methods should be expanded to better describe the type of model used. I assume two random forest models are described here but the terminology Random Forest is not explicitly mentioned.

We have rephrased the M&M section, in particular addressing these comments (see MS and answers to questions below).

Some methodological questions/issues follow:

1. The current approach involves two separate models (presence/absence and abundance random forest) separated by an artificial threshold of 0.5 for the presence probability. Did authors consider applying some kind of zero-altered gamma model (or a similar hurdle/two-part model)? This method could predict both presence and abundance without an arbitrary threshold and potentially integrate uncertainty more naturally. Authors should discuss the rationale for preferring the two-separate-model approach and consider also this different statistical approach for comparison.

We thank the reviewer for this insightful suggestion. We treated this point together with the next point (model uncertainty). Please see combined answer below (point n°2).

2. A related issue is how authors dealt with uncertainty. I understand that in the mechanistic model that is absent and probably could be introduced only by considering instead of point-estimate interval distribution for the parameters. Yet uncertainty could be estimated for the RF model. It appears to me that there is no quantification of uncertainty in the random forest prediction, at least as displayed in the figure. There are some papers eg Zhang, et al. "Random Forest Prediction Intervals." The American Statistician,2019. Doi 10.1080/00031305.2019.1585288 dealing with their computation. Was it assessed and/or what was the rationale to prefer point estimate predictions?

We thank the reviewer for these insightful suggestions regarding the treatment of uncertainty and the use of hurdle-type models.

While we agree that zero-altered or hurdle models provide a unified statistical framework, our approach was designed to explicitly separate the ecological processes governing (i) the occurrence of mosquito activity and (ii) the intensity of oviposition once present. This distinction was central to our study objectives and is supported by the fact that different predictors and lag structures emerged for each process. Additionally, separating the modeling steps avoids issues related to zero-inflated and highly skewed distributions, which are not naturally handled by standard Random Forest regression. Overall, this framework allows us to retain the ecological interpretability of the two-part modeling approach comparable in spirit to hurdle models but more flexible in capturing non-linear relationships. To clarify this point and justify our approach in the manuscript, we added the following sentence in the Methods : This two-step approach was chosen to explicitly separate the environmental drivers of activity onset from those controlling oviposition intensity, rather than modeling both processes jointly, in line with previous studies using similar two-part modeling frameworks (Taconet et al., 2021 ; Mathlouthi et al., 2020 ; Bartholomée et al., 2025).

Regarding uncertainty, we acknowledge that in the original version of the MS, it was not assessed. We agree with the reviewer that this is an important point to treat, and we thank him/her for his suggestion. In response to the reviewer's comment, we have extended our modeling framework to explicitly quantify and propagate uncertainty across both modeling stages.

First, the presence model is now treated probabilistically, and uncertainty is directly represented by the predicted probability of occurrence. Second, for the abundance model, we implemented a quantile random forest (Meinshausen N., 2006) within a leave-one-site-out cross-validation framework, allowing us to derive out-of-sample conditional prediction intervals for positive egg abundance.

Rather than relying solely on a fixed probability threshold, we also computed threshold-free combined predictions as the expected abundance, defined as the product of the probability of presence and the conditional median abundance. To account for uncertainty in both components, we propagated uncertainty to the final prediction using a simulation-based approach, combining the probabilistic occurrence model with the conditional abundance distribution.

This extended framework allows us to retain the ecological interpretability of the two-part modeling approach while providing a coherent and explicit quantification of predictive uncertainty, comparable in spirit to hurdle models but more flexible in capturing non-linear relationships.

The Material and Methods section of the revised manuscript was modified accordingly. Additionally, uncertainty is now represented in Figure 2.

3. To the best of my knowledge, random forest models are quite robust to multicollinearity when predicting but can be affected by it when interpreting the importance of each variable. To avoid this, authors selected a set of variables to reduce multicollinearity based on a procedure involving distance and Pearson correlation. First, since their results highlight specific key drivers, I think more details should be given on what variables were highly collinear and were discarded. Second, variable selection can often be performed naturally within the Random Forest framework (as it ranks variables by importance). Was this considered, and why was the external selection procedure preferred/deemed necessary? Third, The rationale for using distance correlation to capture non-linear associations, only to then resort to Pearson correlation (which is less effective at retaining non-linear associations) in a subsequent selection round, is unclear. Please elaborate on this methodological choice. Finally, the caption for the Cross-Correlation Maps needs to clearly explain the meaning of the black and highlighted borders on some squares.

We thank the reviewer for these constructive comments regarding variable selection and multicollinearity.

On the combined use of distance and Pearson correlation. We thank the reviewer for highlighting this point. We agree that the use of different correlation measures at successive steps could introduce unnecessary methodological inconsistency. In response to this comment, we have revised our variable selection procedure to rely consistently on distance correlation both for identifying relevant predictors and for assessing redundancy among them. This choice ensures that both linear and non-linear dependencies are accounted for throughout the selection process. Please note that this harmonization did not materially affect the results, as the set of selected variables remained unchanged compared to the initial approach based on Pearson correlation.

However, using a single metric throughout the workflow improves the methodological coherence and avoids potential inconsistencies in handling non-linear relationships.

On the identification of collinear variables. We agree that more transparency is needed. In the revised manuscript, we have explicitly reported the variables that were identified as highly collinear (Distance correlation |r| > 0.7). This information was added as a supplementary table (Figure SI4).

On the use of an external variable selection procedure. As reviewer states, while Random Forest models are indeed robust to multicollinearity in terms of predictive performance, correlated predictors can bias the interpretation of variable importance measures, which was a central objective of this study. Since our goal was not only prediction but also the identification and interpretation of key environmental drivers of mosquito activity, we applied an external variable selection procedure to reduce redundancy among predictors. Although embedded variable importance measures in Random Forests can be used for feature selection, they are known to be unstable in the presence of correlated predictors, as importance can be shared or arbitrarily distributed among them. Therefore, we preferred to reduce multicollinearity upstream to ensure more reliable ecological interpretation.

On the interpretation of Cross-Correlation Maps. We agree that the figure caption should be clarified. In the revised version, we have explicitly described the meaning of the graphical elements. Briefly, highlighted cells indicate the lag corresponding to the maximum correlation retained for modeling, while black borders delineate values exceeding a predefined correlation threshold. This clarification was added to the figure explanation.

4. Model evaluation: authors used Spearman and Pearson correlation coefficients between predicted and observed egg counts to assess the ability of the two models to capture the temporal dynamic of oviposition. On one hand I agree that if one model prediction correlate with observation it resembles the temporal trend, on the other its absolute values may be critically different, meaning that the model is biologically far off in depicting the population dynamic. That should be mentioned. Moreover, when using correlation, I am not sure what is the impact of having on one hand the values from RF model set to 0 when the presence probability is below 0.5 and in the other the values of mechanistic probably never reaching zero just approximating it. How would the evaluation results change if the RF model output were instead calculated as (predicted abundance) × (probability of presence)? This alternative output should be considered and discussed.

We thank the reviewer for this insightful comment and agree that correlation-based metrics alone are insufficient to fully assess model performance, as they primarily

capture temporal agreement but not the accuracy of predicted absolute values. In response, we revised our evaluation framework to better reflect the structure of the models and the nature of their outputs.

For the machine learning approach, we now evaluate the two components separately. The presence model is assessed using the area under the ROC curve (AUC) under a leave-one-site-out cross-validation scheme, which quantifies the ability to discriminate between presence and absence. The abundance model, restricted to positive observations, is evaluated using both Spearman correlation and mean absolute error (MAE). While Spearman correlation captures the ability to reproduce temporal dynamics, MAE provides a direct measure of the accuracy of predicted magnitudes.

For the mechanistic model, we retained Spearman correlation as the primary evaluation metric, as its outputs are not directly comparable in magnitude to observed egg counts, preventing the use of error-based metrics such as MAE. To ensure a fair comparison with the machine learning abundance model, we performed two complementary evaluations: (i) using the full time series, including both zero and non-zero observations, and (ii) restricting the evaluation to time steps with observed presence (positive abundance only). This second evaluation allows a more direct comparison with the abundance component of the machine learning model, which is also trained and evaluated on positive observations only.

Finally, regarding the use of thresholded predictions in the machine learning framework, we agree that setting predictions to zero based on a fixed probability threshold may introduce discontinuities. We therefore considered a threshold-free formulation, defining predicted abundance as the expected abundance, i.e. the product of the predicted probability of presence and the conditional abundance prediction. This is consistent with the modifications of our overall modeling approach (see above). This alternative representation yielded consistent results while providing a more continuous and probabilistically coherent output.

These modifications improve both the robustness and the interpretability of the evaluation framework and are now clarified in the revised manuscript.

5. The values from the mechanistic model are averaged and normalized with respect to their highest value to allow comparison with observed ovitrap data. I acknowledge that explicitly modeling the “capture” process, i.e. modeling the number of eggs that will end up laid in an ovitrap requires unavailable (I think) information (e.g. capture radius and capture rate of ovitraps, contribution of skip oviposition behaviour, …). However, the authors’s modeling choice seems to me to implicitly assume that ovitrap data can accurately represent the population dynamics throughout the season and are not characterized by overdispersion.

Two assumptions that are disputed in the literature. Are these assumptions reasonable? Is my reasoning correct? This needs justification or discussion.

There are, in fact, many reasons to believe that ovitraps are not the best way to monitor the exact dynamics of egg-laying—for example, in rainy conditions, they tend to underestimate actual oviposition, because mosquitoes may lay their eggs elsewhere; conversely, during droughts, ovitraps have the opposite effect. On the other hand, ovitraps have numerous advantages; for example, they continuously monitor much larger numbers compared to BG-GAT or BG-Sentinel, whose data are less suited for direct comparison with a mechanistic ODE model. Based on other studies that already directly compare mechanistic models with ovitrap data (Erguler et al., 2016; Tran et al., 2013; Radici et al., 2025), we consider our approach valid, provided we include a qualifying statement in the discussion.

6. I assume that in the model beta depends on temperature also for diapausing eggs and there is a missing (T) in the equation.

Yes, it is the case. We corrected it.

7. The performance metrics show the RF abundance model generally performs better, with the exception of Bayonne, where the mechanistic model shows a significantly higher correlation. There, the correlation for the RF abundance model in is lower (spearman 0.66, pearson 0.23) compared to the mechanistic model (0.88 and 0.92). This discrepancy for Bayonne is worth exploring and/or discussing in consideration of the author statement regarding RF models globally capturing the seasonal trend.

We thank the reviewer for highlighting this point. We agree that the initially observed discrepancy for Bayonne deserved further attention. However, following the revision of our modeling framework—particularly the use of a threshold-free formulation of abundance (expected abundance) and the improved treatment of uncertainty—the performance of the machine-learning model at this site has improved. With this updated framework, the previously observed lower correlation for the RF abundance model in Bayonne is no longer observed, and the machine-learning model now captures the temporal dynamics at this site more consistently. As a result, the discrepancy highlighted by the reviewer is no longer present in the revised results. We have updated the manuscript accordingly and clarified that the machine-learning model consistently captures the seasonal dynamics across sites under the revised framework.

8. Authors assessed the difference in performance by comparing each model to the data and observing that one correlates better than the others. However, the reader is left wondering if the two performances are statistically different or such differences in the correlation values does not provide enough statistical evidence

to reject the hypothesis that both models perform similarly also considering Fig 2.

We thank the reviewer for this important comment. We agree that differences in correlation coefficients alone do not necessarily imply statistically meaningful differences in model performance. To address this point, we complemented our analysis by formally testing the differences in performance between models using a paired bootstrap approach. Specifically, for each site, observations were resampled with replacement and Spearman correlation coefficients were recomputed for both the mechanistic and machine-learning models using the same resampled data. The distribution of the difference in correlation coefficients (machine learning − mechanistic) was then used to derive 95% confidence intervals and associated p-values. This comparison was performed on the abundance component (positive observations only), where correlation-based evaluation is directly comparable across models.

These results are now reported in Supplementary Table SI6. They show that for 3 of the 4 sites, the differences in correlation are not statistically significant, indicating that both models perform comparably despite differences in point estimates. For the Pérols site, the machine-learning model achieves significantly higher correlation than the mechanistic model.

Reviewer #2:

Dear Authors,

I read with interest your manuscript on modelling the oviposition activity of Aedes albopictus in southern France using both a correlative machine learning approach and a mechanistic model. Your work is methodologically robust, the analyses are well executed, and the manuscript is very well written.

However, I would encourage you to reflect on whether the current narrative fully conveys the scientific contribution of the study. While the technical implementation is sound, the novelty and interpretative depth of the findings could be more clearly articulated, particularly with regard to the ecological insights derived from your models.

Let me elaborate:

1. *Scope and Novelty.* I understand and appreciate that this work is intended as a potential starting point for a machine learning-based forecast system for Ae. albopictus activity in southern France. However, as the manuscript currently stands, the novelty of the scientific contribution is not clear. The central finding, that temperature is the main driver of Ae. albopictus oviposition, is well established in the literature given the ectothermic nature of the species (e.g https://doi.org/10.3390/insects9040158, https://doi.org/10.1603/0022-2585-

38.4.548, https://doi.org/10.1186/1756-3305-7-338), and is already embedded in the structure of the mechanistic model you used.

We thank the reviewers for raising this point, which allows us to resolve this ambiguity. As the reviewer noted, temperature is already known to be a key factor in mosquito biology. In fact, it is one of the components included in mechanistic models. However, it is not the only one; in particular, the presence of water (for example, from rain) is *a priori* equally essential. The point of the article is to understand whether, in southern France—an area where this species is non-native—which factor is the limiting (or determining, or in any case the most important) factor in determining its abundance. For example, in areas where rainfall is highly seasonal, it is rainfall rather than temperature that is the factor determining the surge in the abundance of this vector (and the transmission of arbovirus; Polwiang, 2020)

2. *Lag Structure and Temporal Smoothing of temperature*. The incorporation of lagged variables in the correlative model is interesting, and whilst is a solid methodological contribution, I don't think this is the main novelty of the manuscript. In fact, as you note in the manuscript, this has been explored previously. Additionally, I would question whether such a wide lag window for temperature (0–8 weeks) is biologically meaningful. Does this long averaging period dilute the ecological signal? I understand it is the most important variable in your model, but I wonder if the resulting temperature metric seems heavily smoothed and may obscure critical temporal dynamics, such as cumulative heat effects (e.g. GDD), which have been more widely used in phenology models. Is this smoothed temperature variable still interpretable for early warning purposes?

We thank the reviewer for this insightful comment. We agree that the use of lagged climatic variables has been explored previously and do not present it as the primary novelty of this work. Rather, our contribution lies in the systematic identification of relevant temporal scales using cross-correlation analyses and their integration into predictive models.

Regarding the length of the temperature lag window (0–8 weeks), we would like to clarify that this applies to the presence model, which captures the onset and maintenance of mosquito activity, rather than abundance fluctuations. This distinction is important from a biological perspective. The selection of this lag window was data-driven and emerged from cross-correlation analyses, which revealed a broad and sustained association between temperature and the probability of presence over several consecutive weeks, rather than a sharp peak at a specific lag. From an ecological standpoint, the occurrence of eggs reflects the activation of mosquito populations after diapause and the maintenance of suitable conditions for activity. These processes depend on sustained favorable thermal conditions over several weeks, rather than

short-term temperature variability. In this context, the 0–8 week average can be interpreted as a proxy for cumulative thermal suitability, analogous in spirit to growing degree day (GDD) approaches used in phenology, although implemented here in a simpler and data-driven manner. We agree that averaging over several weeks smooths short-term fluctuations. However, for presence/absence, this smoothing is biologically meaningful, as transient temperature spikes are unlikely to trigger or sustain mosquito activity unless they are maintained over time. The broad lag window identified reflects this ecological integration rather than an arbitrary smoothing choice. Finally, the resulting temperature metric remains interpretable for early warning purposes, as it reflects the accumulation of favorable thermal conditions required for the onset of mosquito activity, rather than short-lived anomalies.

To discuss our choices, we have added one sentence in the Discussion section of the revised version of the manuscript: "The relatively long temperature lag identified for presence suggests that mosquito activity is driven by sustained thermal suitability over several weeks, consistent with the progressive activation of populations following diapause".

3. *Relative Humidity*. I think the role of RH in your model deserves a bit of thinking. Although you rightly mention it as an underappreciated variable (but also this paper that is not currently cited https://doi.org/10.1111/ele.14228) , its temporal and spatial volatility, especially at a weekly scale, raises questions about its reliability in predictive models. Given also your own reservations expressed in L258–261, is it appropriate to retain RH in the final model?

We thank the reviewer for this important comment. We agree that relative humidity is a more difficult variable to interpret mechanistically than temperature, both because of its strong context dependence and because of its temporal variability. We also agree that its role in mosquito ecology has often been underappreciated, and we have now added the suggested reference on this point. Brown et al. (2023) emphasize that humidity can interact with temperature in complex ways and deserves greater attention in mosquito-borne disease modeling, while also highlighting that its effects are not straightforward and may vary across systems.

In our study, RH was retained not because we interpret it as a primary driver, but because it emerged from the same data-driven selection procedure as the other predictors and was not redundant with the retained temperature variables after collinearity filtering. In addition, its contribution remained secondary compared with temperature in both the variable-importance and partial-dependence analyses, which is consistent with our interpretation in the manuscript.

We agree that RH should be interpreted cautiously. For this reason, we do not present it as a robust standalone determinant of mosquito activity, but rather as a secondary

predictor that may capture part of the local atmospheric context not fully represented by temperature and rainfall alone. We have revised the text in the Discussion to make this limitation clearer and to avoid over-interpreting its ecological meaning. In particular, we now emphasize that RH improved the predictive model as a statistical covariate, while its mechanistic role remains uncertain in our study system.

We additionally performed a sensitivity analysis excluding RH from the final model. This did not materially change the main conclusions, in particular the dominant role of temperature, but slightly reduced predictive performance. We therefore retained RH in the final specification as a secondary, data-supported predictor.

4. *Rainfall.* I found the precipitation results interesting, particularly the difficulty in establishing a consistent signal, a finding that mirrors the challenges in other studies. This reinforces the complexity of linking rainfall to oviposition at coarse spatio-temporal scales and deserves greater discussion. Additionally we might ask ourself the question if these correlative approaches are the right statistical methods to infer the relationship between rainfall and critical aspects of the species biology. What does the mechanistic model have to say about it? Can you produce a figure similar to Fig.5 but for the effect of precipitation on different life-history trait?

We thank the reviewer for this suggestion. We added this analysis the SI (Figure 8).

5. *Interpretative Focus*. A key unresolved issue is whether the manuscript aims primarily to develop a predictive tool or to gain ecological insight into Ae. albopictus oviposition dynamics. If it is the former, I would expect some form of out-of-sample validation or spatial extrapolation (e.g., using datasets like VectAbundance mentioned in the manuscript). If it is the latter, then results like those in Figures 4 and 5, which potentially reveal biological or climatic thresholds, deserve more analytical space and interpretative emphasis.

The main purpose of this article is, in fact, to contribute to our ecological understanding of the thermal biology of this invasive species. For the co-first authors, it is also the first step in a line of thinking aimed at developing an early warning model, which is why we believe it is appropriate to mention this aspect in the discussion. In light of the revisions made in response to the comments from the three reviewers, we are confident that the new version of the manuscript is more effective in fulfilling its primary purpose (and that it addresses the reviewer's concerns regarding the space allocated to the analysis of the results and the interpretative emphasis).

I believe this is a high-quality manuscript with the potential to make a meaningful contribution, particularly if the narrative better emphasises either its predictive

utility or the biological insights it provides. I hope these comments are helpful in refining the manuscript.

Minor Comments

Introduction

• L63: The phrasing "models assume climate as the main driver" is problematic: mechanistic models are built on this premise, but it's not an assumption in all contexts. See e.g. Reinhold et al. (2018) for clarification.

We thank the referee for pointing out this ambiguity. However, unless there are errors in uploading the manuscript, in the version we submitted, the phrasing is "Ultimately, most models assume – either implicitly or explicitly – that the seasonal dynamics of *Ae. albopictus* depend on weather conditions." To us, this sentence looks correct.

• L64: Consider rephrasing "eggs to pupae" as "aquatic stages" to improve clarity.

Thanks, we corrected it.

• L75–78: The mention of arbovirus risk mapping seems out of place in this section, which is otherwise focused on phenology. Consider moving this to the end of the introduction or to the discussion.

Thanks, we removed this mention and integrated this concept in the discussion.

Methods

• Have you tested whether an ensemble model (e.g. weighted average of ML and mechanistic models) would improve performance?

We thank the reviewer for this suggestion. While ensemble approaches combining machine-learning and mechanistic models may indeed improve predictive performance, the primary objective of this study was not to maximize prediction accuracy but rather to better understand and compare the environmental drivers and dynamics captured by each modeling framework. In this context, we focused on analyzing the behavior and interpretability of each approach separately, rather than combining them into a single predictive model. Exploring ensemble strategies would be a valuable direction for future work, particularly in an operational forecasting perspective, but was beyond the scope of the present study.

• L368: Please define CCM acronym.

Done.

• L390: The threshold seems somewhat arbitrary: did you consider optimising it based on model sensitivity or another metric?

We have revised the overall machine-learning modeling framework (see Methods section of the revised version), which is now based on a threshold-free formulation, defining predicted abundance as the expected abundance, i.e. the product of the predicted probability of presence and the conditional abundance prediction.

Results

• L94–105: Present Figure 1 before Figure 2 for logical consistency.

Actually, we would prefer to keep Figure 1 – which presents the data – before Figure 2 – which presents modelling outputs. We acknowledge there was an error quoting Fig. 1 in the manuscript, which we now corrected.

• Figure 2: In sites sampled every two weeks, how do you determine the true onset/offset of the season? What if the activity peak falls between sampling dates?

We thank the reviewer for this important point. We agree that biweekly sampling introduces uncertainty in the exact timing of seasonal onset, offset, and peak activity, as these events may occur between two sampling dates. In our study, these metrics are therefore defined operationally based on the first and last observed detections, and the highest observed values, rather than as exact biological transition points. However, our modelling framework does not rely on the precise timing of these events, but rather on broader temporal dynamics captured at weekly to multi-week scales. In particular, both the mechanistic and machine-learning models integrate environmental information over several weeks, which reduces sensitivity to short-term variability occurring between sampling dates. As a result, while some temporal smoothing is inherent to the sampling design, it is consistent across sites and does not affect the main conclusions of the study. We have clarified this limitation in the revised manuscript with the following sentence : “Given the biweekly sampling design at some sites, onset, offset, and peak activity should be interpreted as observed rather than exact dates, with an uncertainty of up to one sampling interval.”

• Table 1: Please include a measure of uncertainty or variability (e.g. confidence intervals) for the correlation coefficients.

The p-value was provided in Table SI5.

• Figure 3: Increase label font size for readability.

Done.

• L145: Can you estimate and report at which temperature the inflection point is located?

Between 12.9 and 13.1 depending on the site.

• Figure 4: The "no activity" symbol might be misinterpreted as indicating vector control, consider a more neutral symbol for winter.

We have modified the symbol across the whole manuscript.

• Consider adding distributions (e.g. quantiles) of the observed response variable by site to contextualise the underestimation of peaks in the correlative model.

We have added the 5th–95th percentile interval derived from the quantile regression forest.

Discussion
• L202–204: This sentence is unclear, please revise for clarity.

Thanks, we revised the sentence to increase clearness.

• L229–230: Again, the shift to arbovirus risk seems abrupt. I would recommend consolidating all disease-related discussion at the end to maintain narrative flow.

We understand the point raised by the reviewer. Actually, in this case we would prefer keep this quotation, since it is related to the title of the paragraph ("Warm temperatures facilitate longer and more intense activity seasons of *Aedes albopictus* in a temperate climate"), at the end of it. We slightly modified the text to make the shift less abrupt.

• Consider adding a limitation regarding the relatively short time series and the limited number of surveillance sites.

We added it in the paragraph "Limitations of the study".

• Ensure species names are italicised consistently in the references.

Done, thanks (in the untracked cleaned version).

Reviewer #3:

Thank you for the opportunity to review this interesting piece of work. It provides an important contribution to the understanding of the ecology and climate suitability of Aedes Albopictus in Southern France. The authors used ovitraps collection data and climatic factors. They analyzed the most contributing weather variables to eggs presence and abundance. To do so, they determined the most important variables and lags through a bivariate correlation test. They used the selected variables and lags to build a machine-learning model (random forest) to predict eggs presence and abundance. They additionally analyzed the interannual variability of eggs counts and

their relation with climate-dependent demography parameters from a mathematical model for Ae. Albopictus.

Strengths:
The authors' study covers the important and concerning topic of the spread of the Asian tiger mosquito in Europe. The authors come up with a useful machine-learning based model that deliver impressive predictions, mainly based on temperature. This model can be very useful for a better understanding of mosquito population dynamics, as well as for preparedness against mosquitoes nuisance and infectious diseases outbreaks.

Weaknesses:
Major comments:

1. **The selection of lagged weather variables is based on bivariate correlation analyses. This approach is known to be potentially misleading in settings where predictors are highly correlated, as is typically the case for climatic variables. A variable may appear influential at a given lag due to confounding with another correlated variable or lag, rather than reflecting an independent effect. On the contrary, bivariate correlation analyses may fail to identify variables that act primarily at shorter time scales when a strong seasonal signal dominates the response. In such cases, an influential predictor may appear weak or unimportant because its effect is masked by an externally imposed seasonal pattern. This may be the case, for example, for rainfall, which typically exhibits less pronounced annual seasonality than temperature but may still contribute meaningfully to oviposition dynamics at short temporal scales. Consequently, the selected variables and lags may not be identifiable, and their selection should be treated with caution. This limitation should be explicitly acknowledged and discussed.**

We thank the reviewer for this important comment and fully agree that the use of bivariate correlation analyses for lag selection has inherent limitations, particularly in the presence of correlated predictors and strong seasonal signals.

In our approach, cross-correlation analyses were used as an exploratory step to identify candidate variables and plausible temporal scales of association. We acknowledge that such analyses do not isolate independent effects and may reflect shared seasonality or correlations between predictors. In particular, variables with weaker seasonal structure, such as rainfall, may be underrepresented in this step despite potentially important short-term effects.

To mitigate these limitations, the bivariate selection was followed by a multivariate modeling step, in which variables were jointly evaluated within a Random Forest framework. This allowed us to account for interactions and non-linear relationships

between predictors. In addition, collinearity among predictors was explicitly addressed prior to model fitting to reduce redundancy.

We agree that the resulting variable and lag selection should be interpreted with caution, and we have revised the Discussion section of the manuscript ("Limitations of the study") to explicitly acknowledge that the identified associations reflect combined effects of correlated climatic drivers and may not correspond to independent causal contributions.

2. **It is unclear why the selected predictors are defined as averages across multiple lag periods, and how this choice follows from the preceding variable and lag selection procedure. This lack of clarity also weakens the interpretation of the statement in the Discussion that "Average temperature of the previous 9 weeks is by far the main determinant of the presence of eggs, and of the previous 5 for their abundance." As the predictors are defined as averages over selected lag windows, it is difficult to disentangle whether these specific lag durations emerge from the data or are, at least in part, imposed by the modeling choices. Clarifying how these lag windows were identified is therefore important for supporting this conclusion.**

We thank the reviewer for this comment and agree that the link between the lag selection procedure and the construction of averaged predictors required clarification. Lag selection was first performed using cross-correlation analyses across a range of individual lags. Rather than identifying a single optimal lag, these analyses consistently revealed broad peaks of high correlation spanning several consecutive weeks. The use of averaged predictors over these lag windows was therefore intended to capture these sustained temporal responses and reduce sensitivity to noise at individual lags. Importantly, the lag windows used in the final models were not arbitrarily defined but were based on the extent of these correlation plateaus. However, we acknowledge that averaging across lag windows introduces a degree of smoothing, and that the precise duration of these windows (e.g. 0–8 or 1–5 weeks) should not be interpreted as sharply defined biological thresholds. We have revised the manuscript (Methods + Discussion) to clarify this point and to emphasize that these predictors reflect cumulative or sustained environmental conditions rather than effects at specific time lags.

3. **The number of traps in every site influences the egg counts. This is not clear how this aspect is addressed in the methodology.**

In fact, each site has a number of ovitraps that remains relatively constant over time and is of the same order of magnitude across sites, though not necessarily the exact same number. For this reason, for each site and each date, the average number of eggs observed during that week is calculated. According to the central limit theorem, this indicator tends to be more accurate as the number of ovitraps increases, in a non-linear

manner (the standard deviation increases with the square root of the number of ovitraps). Since the number of ovitraps varies little from area to area, we have neglected this difference.

Minor comments:

1. In the Author summary, the statement that the modelling approaches "qualitatively represent the observed trends" is vague and difficult to evaluate.

We replaced "qualitatively represent the observed trends" with “reproduce the observed trends”.

2. In the introduction, it is unclear what is actually the research gap, the aim of the paper and how they relate. Stated research gap: "Despite growing research effort to clarify the role of weather determinants of Ae. albopictus activity (15–20), local authorities, public health services and mosquito control agencies often lack of solid scientific evidence to support their policies. These are ultimately jeopardized by climate changes, expected to affect the seasonality of this vector (21)." Do the authors want to provide an early warning system? Or to add on the knowledge of climatic effect on Ae. Albopictus populations? The impact of climate change is not addressed in the paper (but discussed).

We would like to thank the reviewer for raising this point, which has allowed us to refine the article’s definition. The aim is to enhance understanding on *Ae. albopictus*; however, since we are currently working on developing an early warning system, we would like to explore this point further in the discussion (as a possible and necessary direction for the development of this work).

3. Throughout the manuscript, the notion of machine learning is associated with statistical model (for instance, "We model oviposition dynamics using both weather-driven (machine learning-based) statistical and mechanistic approaches [...]"). This terminology is potentially misleading, as the methods employed (e.g. random forest–type algorithms) are predictive, data-driven models that do not constitute statistical models in the inferential sense.

We thank the reviewer for this important clarification. We have revised the manuscript to harmonize the terminology throughout and now consistently refer to these approaches as *machine learning models* rather than *statistical models*. While our primary objective is explanatory rather than predictive, we chose machine learning methods due to their ability to capture complex, non-linear interactions among environmental variables, as well as the increasing use of interpretable machine learning approaches in ecological and biological studies. This clarification improves the conceptual consistency of the manuscript and avoids potential confusion regarding the nature and purpose of the modeling framework.

4. The structure of the Material and Methods section and the related Results is not straightforward. The manuscript would gain in clarity if the interannual variation topic was entirely grouped after the impact of weather variables.

We understand the remark by the reviewer. Unfortunately, we were unable to find a compromise that was 100% satisfactory given the need to (a) present the data in the first section (and thus, inevitably, analyze them); and (b) prioritize the results derived from the statistical model analysis. In the version we are resubmitting, we attempt to revisit the topic of year-over-year comparisons of oviposition at the end, and we hope it will be clearer.

References

Meinshausen, N. (2006). Quantile Regression Forests. Journal of Machine Learning Research, 7, 983–999.

Taconet, P., Porciani, A., Soma, D.D. et al. Data-driven and interpretable machine-learning modeling to explore the fine-scale environmental determinants of malaria vectors biting rates in rural Burkina Faso. Parasites Vectors 14, 345 (2021). https://doi.org/10.1186/s13071-021-04851-x

Mathlouthi W, Larocque D, Fredette M. Random forests for homogeneous and non-homogeneous Poisson processes with excess zeros. Stat Methods Med Res. 2020;29(8):2217–37.

Bartholomée, C., Taconet, P., Mercat, M., Grail, C., Bouhsira, E., Fournet, F., & Moiroux, N. (2025). Investigating the role of urban vegetation alongside other environmental variables in shaping Aedes albopictus presence and abundance in Montpellier, France. PLOS One, 20(11), e0335793. https://doi.org/10.1371/journal.pone.0335793

Erguler, K., Smith-Unna, S. E., Waldock, J., Proestos, Y., Christophides, G. K., Lelieveld, J., & Parham, P. E. (2016). Large-scale modelling of the environmentally-driven population dynamics of temperate Aedes albopictus (Skuse). PloS one, 11(2), e0149282. https://doi.org/10.1371/journal.pone.0149282

Tran, A., L'ambert, G., Lacour, G., Benoît, R., Demarchi, M., Cros, M., ... & Ezanno, P. (2013). A rainfall-and temperature-driven abundance model for Aedes albopictus populations. *International journal of environmental research and public health*, *10*(5), 1698-1719. https://doi.org/10.3390/ijerph10051698

Radici, A., Hammami, P., Cannet, A., L'Ambert, G., Lacour, G., Fournet, F., ... & Caminade, C. (2025). Aedes albopictus is rapidly invading its climatic niche in France:

Wider implications for biting nuisance and arbovirus control in Western Europe. Global Change Biology, 31(8), e70414. https://doi.org/10.1111/gcb.70414

Polwiang, S. (2020). The time series seasonal patterns of dengue fever and associated weather variables in Bangkok (2003-2017). BMC infectious diseases, 20(1), 208. https://doi.org/10.1186/s12879-020-4902-6
