# Supplementary material for: Environmental and demographic determinants of Aedes albopictus seasonal activity in southern France: A modeling study
Source: PLoS Comput Biol. 2026 Sep 10;22(9):e1014799. doi: 10.1371/journal.pcbi.1014799 (PMC13588507; doi:10.1371/journal.pcbi.1014799)
Supplement: Supplementary file 2 [file pcbi.1014799.s004.pdf]

Dear Editor,

This letter accompanies the revision of the manuscript “Environmental and demographic determinants of *Aedes albopictus* seasonal activity in southern France: a modeling study”. Below we provide the point-by-point responses (in green) to the reviewer’s comments.

Sincerely,

The authors

Point by point reply to reviewers

Editor comments:

1) We have noticed that you have uploaded Supporting Information files, but you have not included a list of legends. Please add a full list of legends for your Supporting Information files after the references list.

Done (L. 735-756 of the tracked file).

2) When completing the data availability statement of the submission form, you indicated that you will make your data available on acceptance. We strongly recommend all authors decide on a data sharing plan before acceptance, as the process can be lengthy and hold up publication timelines. Please note that, though access restrictions are acceptable now, your entire data will need to be made freely accessible if your manuscript is accepted for publication. This policy applies to all data except where public deposition would breach compliance with the protocol approved by your research ethics board. If you are unable to adhere to our open data policy, please kindly revise your statement to explain your reasoning and we will seek the editor's input on an exemption. Please be assured that, once you have provided your new statement, the assessment of your exemption will not hold up the peer review process.

Thanks for the reminder. We uploaded all data and code used for the analysis on a Zenodo repository available at: https://zenodo.org/records/21680620 (L. 534-535)

3) We have amended your Competing Interest statement to comply with journal style. We kindly ask that you double check the statement and let us know if anything is incorrect.

The new Competing Interest statement works for us.

Reviewers' comments:

Reviewer #1:

Despite I partially disagree with a couple of responses provided by authors:

1. “This distinction [separating explicitly the ecological processes] was central to our study objectives and is supported by the fact that different predictors and lag structures emerged for each process” zero-altered and hurdle models allows modeling with different covariates the different processes, at least in a Bayesian framework, see Alain Zuur The World of Zero-Inflated Models: Vol 1-3

We thank the reviewer for this remark. We agree that zero-altered and hurdle models provide an elegant statistical framework for modeling occurrence and positive abundance with distinct covariates and ecological processes. However, to the best of our knowledge, there is currently no widely established machine-learning equivalent that combines the flexibility of Random Forests with a unified hurdle or zero-altered formulation. Consequently, we adopted a two-stage machine-learning framework consisting of a Random Forest classifier for presence and a Quantile Regression Forest for positive abundance. This approach allowed us to model the two processes independently while retaining the advantages of machine-learning methods, including the ability to capture complex non-linear relationships and to interpret each process separately. We have revised the manuscript to clarify this rationale, by citing three studies on the subject (1–3; L. 406-413) .

2. “continuously monitor much larger numbers compared to BG-GAT or BG-Sentinel, whose data are less suited for direct comparison with a mechanistic ODE model” there are various articles using adult data to fit ODE models, see a recent example in Virgillito et al (2025) doi.org/10.1038/s43856-025-00983-8

We acknowledge that other studies used adult data to fit ODE models. What we meant is that ODEs are supposed to represent fully deterministic phenomena, while entomological counts are subject to various sources of stochasticity (a mosquito may or may not be trapped; observers may miscount mosquitoes…). However, the impact of stochasticity is reduced when dealing with large numbers, and ovitraps usually provide higher abundances than adult traps because of (a) pyramidal age structure (within the same population, eggs are generally more abundant than adults) and (b) longer trapping time (1-3 weeks for ovitraps, few days usually for adult traps, which usually are battery-powered, and need a source of carbon dioxide).

I think they fully addressed the issues I raised and have no further revision requests

Reviewer #2:

Dear Authors,

Thank you for the revised manuscript and for the thorough point-by-point responses to the reviewers' comments. The authors have addressed the concerns raised in the previous round satisfactorily, and I consider the manuscript substantially improved. I am in principle supportive of publication, subject to three minor points that I'd ask you to address.

1. Season extension claim. The manuscript argues that warmer springs and autumns are likely to extend the active season of Ae. albopictus under climate change. This is a mechanistically coherent argument grounded in the temperature dependence of the species' life-history traits. However, the two-year dataset underlying this study is insufficient to provide empirical support for a directional trend in season length. With only two breeding seasons, any apparent difference in onset or offset dates falls within the range of natural interannual variability and cannot be distinguished from it statistically. The authors should clarify explicitly that the season extension argument is a mechanistic inference rather than an empirical finding, and qualify the relevant statements accordingly.

We agree that we this aspect need to be better contextualized with respect to the existing literature on the link between global warming and seasonality. As the reviewer states, this claim is a (mechanistic) consequence of both (a) thermal dependency of tiger mosquitoes and (b) global warming, and in this study we focus only on the first point. We modified it (L. 236) by adding references to two recent studies (4,5) on the subject.

2. Definition of the ML response variable. The manuscript defines the response variable of the machine learning abundance model as the daily number of eggs per ovitrap (Methods section). This is incorrect. Since ovitraps were sampled every one to two weeks, the recorded egg count represents a cumulative total over the active period of the trap, not a daily value. The response variable should be described as the cumulative number of eggs per trap per sampling period, or equivalently as the mean egg count per sampling interval. The current phrasing risks misleading readers about the temporal resolution and magnitude of the modelled quantity.

Actually, for each observation, we divided the egg count for the duration of the sampling event; therefore, the response variable is truly a daily mean. The same we did for the mechanistic comparison (L. 463-465). We acknowledge that this is not completely understandable from the text, which we modified (L. 399-401)

3. Wilcoxon test and temporal autocorrelation. The Wilcoxon test is used to compare the distribution of ovitrap records between 2023 and 2024 at each site. Within-season observations at the same site are temporally autocorrelated, which violates the independence assumption of the test and inflates the effective sample size. The authors should acknowledge this limitation in the Discussion, as it further qualifies the strength of the interannual comparisons on which some of the conclusions rest.

We thank the reviewer for having pointed out this limitation. If we interpret correctly his/her point, however, the significative difference of a variable between two intra-annual samplings is somehow even stronger, due to temporal autocorrelation, which would spontaneously lead to an overestimation of non-significant tests. We acknowledge these limitations in a paragraph in the Discussion (L. 325-330)

Once these aspects are addressed, I think the manuscript can be accepted for publication.

We thank the reviewer for his/her encouraging feedback.

Reviewer #3:

I would like to thank the authors for carefully reviewing the manuscript following my comments, and for the clear point-by-point responses. The manuscript has substantially gained in clarity, with the limitations now better stated and the methodology clarified. From my perspective the manuscript is now ready for publication as it is.

We thank the reviewer for his/her positive feedback.

References:

1. Taconet P, Porciani A, Soma DD, Mouline K, Simard F, Koffi AA, et al. Data-driven and interpretable machine-learning modeling to explore the fine-scale environmental determinants of malaria vectors biting rates in rural Burkina Faso. Parasites Vectors. 2021 Jun 29;14(1):345. doi:10.1186/s13071-021-04851-x

2. Mathlouthi W, Larocque D, Fredette M. Random forests for homogeneous and non-homogeneous Poisson processes with excess zeros. Stat Methods Med Res. 2020 Aug;29(8):2217–37. doi:10.1177/0962280219888741 PubMed PMID: 31762374.

3. Bartholomée C, Taconet P, Mercat M, Grail C, Bouhsira E, Fournet F, et al. Investigating the role of urban vegetation alongside other environmental variables in shaping Aedes albopictus presence and abundance in Montpellier, France. PLOS ONE. 2025 Nov 12;20(11):e0335793. doi:10.1371/journal.pone.0335793

4. Del Lesto I, De Liberato C, Casini R, Magliano A, Ermenegildi A, Romiti F. Is Asian tiger mosquito (Aedes albopictus) going to become homodynamic in Southern Europe in the next decades due to climate change? Royal Society Open Science. 2022 Dec 14;9(12):220967. doi:10.1098/rsos.220967

5. Virgillito C, Longo E, De Marco CM, Gentile C, Micocci M, Topalidis C, et al. Cross-sectional entomological data reveals an increased risk of arboviral transmission in a year of record-breaking heat in Southern Europe. Commun Med. 2025 Jul 23;5(1):307. doi:10.1038/s43856-025-00983-8
