# Supplementary material for: Environmental and demographic determinants of Aedes albopictus seasonal activity in southern France: A modeling study
Source: PLoS Comput Biol. 2026 Sep 10;22(9):e1014799. doi: 10.1371/journal.pcbi.1014799 (PMC13588507; doi:10.1371/journal.pcbi.1014799)
Supplement: Supplementary file 3 [file pcbi.1014799.s001.docx]

# **Appendix for:**

# Environmental and demographic determinants of *Aedes albopictus* seasonal activity in southern France: a modeling study

Paul Taconet*^1,4^, Andrea Radici*^1^, Guillaume Lacour^2^, Antoine Mignotte^2^, Pachka Hammami^3^, Didier Fontenille^1^

^1^MIVEGEC (Université de Montpellier, IRD, CNRS), F-34090 Montpellier, France; ^2^Altopictus, F-34470 Pérols, France ; ^3^ASTRE (CIRAD, INRAE, Université de Montpellier), F-34398 Montferrier-sur-Lez, France ^4^ TETIS (INRAE, AgroParisTech, CIRAD, CNRS, Univ Montpellier, Montpellier, France)

*co-first authors

## Additional materials

### Metelmann model parameters

In the modelling framework established by Metelmann et al., (2019) both the carrying capability $K_{x}$ and the hatching $h_{x}$ of a site $i$ at time $t$ depend on host density $H_{x}$ and rainfall $r_{x}$:

$K_{x}=\lambda\frac{1-\alpha_{evap}}{1-{\alpha_{evap}}^{t}}\sum_{x=1}^{t} {\alpha_{evap}}^{\left( t-x \right)}(\alpha_{rain}r_{ix}+\alpha_{dens}H_{ix}$*)*

$$h_{x}=\left( 1-\varepsilon_{rat} \right)\frac{\left( 1+\varepsilon_{0} \right)e^{-\varepsilon_{var}\left( r_{x}-\varepsilon_{opt} \right)^{2}}}{e^{-\varepsilon_{var}\left( r_{x}-\varepsilon_{opt} \right)^{2}}+\varepsilon_{0}}+\varepsilon_{rat}\frac{\varepsilon_{dens}}{\varepsilon_{dens}+e^{-\varepsilon_{fac}H_{x}}}$$

Note that, in the main text, we have indicated the physical quantity of each environmental driver, without distinguishing among different functional forms. For example, both adult mortality
$\mu_{A}(\overline{T})$ and survival probability of diapausing eggs $\gamma(T_{min\left( DJF \right)})$ depends on temperature, but the former refers to its daily average, while the latter depends on the minimum value in the previous winter.

Here we present the table of the values of the parameters of the abovementioned equations and those of the compartmental model presented in the main text, with the corresponding source:

Table A – Metelmann model parameters

| **Parameter** | **Meaning** | **Value/formula** | **Source** |
| --- | --- | --- | --- |
| ${CTT}_{S}$ | critical temperature over one week in spring (°C ) | $11.0$ | (1) |
| ${CPP}_{S}$ | critical photoperiod in spring (hours) | $11.25$ | (1) |
| $\sigma(T,P)$ | spring hatching rate (day^-1^) | $\left\{ \begin{aligned} 0ifT_{7}<{CTT}_{S}\vee P<{CPP}_{S} \\ 0.1otherwise \end{aligned} \right.$ | (1) |
| ${CPP}_{A}(L)$ | critical photoperiod in autumn (hours) | $10.058+0.08965L$ | (1) |
| $\omega(P)$ | fraction of eggs going into diapause | $\left\{ \begin{aligned} 0ifP<{CPP}_{A}\vee day<183 \\ 0.5otherwise \end{aligned} \right.$ | (1) |
| $\delta_{E}$ | normal egg development rate (day^-1^) | $1/{7.1}$ | (1) |
| $\delta_{J}(T)$ | Juvenile development rate (day^-1^) | $1/(83.85-4.89T+0.08T^{2})$ | (1) |
| $\delta_{I}(T)$ | first pre-blood meal rate (day^-1^) | $1/(50.1-3.574T+0.069T^{2})$ | (1) |
| $\mu_{E}$ | egg mortality rate (day^-1^) | $-ln(0.955e^{-0.5{(\frac{T-18.8}{21.53})}^{6}})$ | (1) |
| $\mu_{J}$ | juvenile mortality rate (day^-1^) | $-ln(0.977e^{-0.5{(\frac{T-21.8}{16.6})}^{6}})$ | (1) |
| $\mu_{A}(\overline{T})$ | adult mortality rate (day^-1^) | $-ln(0.677e^{-0.5{(\frac{\overline{T}-20.29}{13.2})}^{6}}0.069\overline{T}^{0.1})$ | (1) |
| $\gamma(T_{min\left( DJF \right)})$ | survival probability of diapausing eggs (winter^-1^) | $0.93e^{-0.5{(\frac{T_{min\left( DJF \right)}-11.68}{15.67})}^{6}}$ | (1) |
| $\beta(T)$ | egg laying rate (day^-1^) | $\left\{ \begin{aligned} 33.2e^{-0.5{(\frac{T-70.3}{14.1})}^{2}}{(38.8-T)}^{1.5}ifT\leq38.8 \\ 0otherwise \end{aligned} \right.$ | (1) |
| $\lambda$ | capacity parameter (larvae days ha^-1^) | ${10}^{6}$ | (1) |
| $\alpha_{evap}$ | Normalization parameter evaporation | $0.9$ | (1) |
| $\alpha_{dens}$ | Normalization parameter of host density (km²) | ${10}^{-5}$ | (1) |
| $\alpha_{rain}$ | Normalization parameter of rain (mm^-2^) | ${10}^{-2}$ | (1) |
| $\varepsilon_{rat}$ | Proportion of hatching due to rain | $0.2$ | (1) |
| $\varepsilon_{0}$ | Correcting additive parameter for rain-related hatching | $1.5$ | (1) |
| $\varepsilon_{var}$ | Stretching parameter for rain-related hatching (mm^-2^) | $0.05$ | (1) |
| $\varepsilon_{opt}$ | Correcting additive parameter for human-induced hatching (mm) | $8$ | (1) |
| $\varepsilon_{dens}$ | for rain-related hatching | ${10}^{-2}$ | (1) |
| $\varepsilon_{fac}$ | Stretching parameter for human-induced hatching (km^-2^) | ${10}^{-2}$ | (1) |

The model scheme is represented here:

Fig A – Layout of the weather-driven Metelmann model describing Ae. albopictus dynamics, modified from (2) by removing unnecessary details (R_0_, suitability, adult density).

### Physical quantities in models

In the following table, we report the explicit definition of the weather and environmental variables:

Table B – Weather variables and specifications

| **Weather and environmental variable** | **Meaning** |
| --- | --- |
| $T$ | Instantaneous temperature (°C) |
| $\overline{T}$ | Mean daily temperature (°C) |
| $T_{7}$ | Mean temperature of the last week (°C) |
| $T_{min\left( DJF \right)}$ | Lowest winter (December-January-February) temperature (°C) |
| $R$ | Daily rainfall (mm) |
| $H$ | (Human) Host density (hab/km^2^) |
| $P$ | Photoperiod (in hour) |
| $L$ | Latitude (°) |

### Ovitrap collection details

In the following table, details about the ovitrap records samplings. In rare cases in which the average temperature was missing, it was computed as the mean of the maximal and minimal daily temperature.

Table C – Summary about ovitrap and weather recording per site

| **Site** | **Number of ovitraps** | **Mean number of active ovitraps per week** | **Series length (number of collection dates)** | **Years** | **Corresponding Météo France weather station** | **Human density (km^-2^, average from GPW)** |
| --- | --- | --- | --- | --- | --- | --- |
| Pérols | 20 | 19.1 | 64 | 2023-2024 | Montpellier-aéroport | 1013.7 |
| Murviels-les-Montpellier | 30 | 27.7 | 43 | 2023-2024 | Montarnaud | 102.1 |
| Saint-Médard-en-Jalle | 20 | 19.9 | 47 | 2023-2024 | Bordeaux-Merignac | 991.4 |
| Bayonne | 20 | 20.5 | 41 | 2023-2024 | Biarritz-Pays-Basque | 826.0 |

Table D – Extended summary of ovitrap and weather data per site, year and season

| Site | Year | Season | Weather | | Entomology | | |
| --- | --- | --- | --- | --- | --- | --- | --- |
|  |  |  | Average T (°C) | Cumulated rainfall in season (mm) | Mean abundance (egg/ovitrap) | Begin of activity (week) | End of activity (week) |
| Pérols | 2023 | Spring | 21.1 | 85.4 | 19.6 | 19 | 46 |
|  |  | Summer | 25.2 | 15.9 | 30.7 |  |  |
|  |  | Autumn | 20.3 | 54.3 | 43.7 |  |  |
|  |  | Nov-> Ap | 10.6 | 105.7 | 0.3 |  |  |
|  | 2024 | Spring | 19.4 | 105.4 | 14.3 | 20 | 51 |
|  |  | Summer | 25.6 | 26.6 | 38.5 |  |  |
|  |  | Autumn | 18.7 | 118.1 | 36.0 |  |  |
|  |  | Nov-> Ap | 11.0 | 329.6 | 0.8 |  |  |
| Mur.-les-Montpellier | 2023 | Spring | 20.0 | 153.6 | 23.7 | 18 | 48 |
|  |  | Summer | 24.9 | 13.6 | 40.9 |  |  |
|  |  | Autumn | 18.9 | 159.3 | 28.8 |  |  |
|  |  | Nov-> Ap | 9.6 | 120.8 | 0.5 |  |  |
|  | 2024 | Spring | 18.5 | 140.4 | 8.3 | 19 | 50 |
|  |  | Summer | 25.1 | 29.4 | 28.4 |  |  |
|  |  | Autumn | 17.3 | 234.1 | 16.7 |  |  |
|  |  | Nov-> Ap | 10.0 | 428.2 | 0.3 |  |  |
| Bayonne | 2023 | Spring | 18.7 | 161.2 | 6.0 | 19 | - |
|  |  | Summer | 21.4 | 148.1 | - |  |  |
|  |  | Autumn | 20.1 | 355.2 | - |  |  |
|  |  | Nov-> Ap | 11.1 | 1,072.8 | 0.0 |  |  |
|  | 2024 | Spring | 16.7 | 387.8 | 3.5 | 17 | 51 |
|  |  | Summer | 21.3 | 70.2 | 22.8 |  |  |
|  |  | Autumn | 17.3 | 536.7 | 20.2 |  |  |
|  |  | Nov-> Ap | 11.7 | 824.7 | 0.4 |  |  |
| St.-Médard-en-Jalle | 2023 | Spring | 19.8 | 156.0 | 8.2 | 19 | - |
|  |  | Summer | 22.0 | 55.6 | - |  |  |
|  |  | Autumn | 19.4 | 280.9 | - |  |  |
|  |  | Nov-> Ap | 9.8 | 693.9 | 0.0 |  |  |
|  | 2024 | Spring | 17.4 | 219.6 | 3.6 | 20 | 49 |
|  |  | Summer | 22.0 | 60.9 | 32.2 |  |  |
|  |  | Autumn | 16.4 | 206.7 | 16.4 |  |  |
|  |  | Nov-> Ap | 10.3 | 529.6 | 0.1 |  |  |

### Cross correlation maps

In the following figures, we show the cross correlation maps between lagged environmental drivers and ovitrap measurements. In Fig B, we show the correlation for the presence-absence model, while in Fig C for the continuous abundance model. The red-bordered square indicates the time lag interval that showed the highest Distance correlation coefficient with the meteorological variable (the associated time lag interval and correlation coefficient are reported on the top-left corner of the CCM). The black-bordered squares indicate correlations close to the highest observed correlation (i.e. less than 10% of difference). Gray-filled squares indicate low or non-significant correlations (p-value > 0.2 or coefficient > 0.1).

Fig B – Cross correlation maps for each site (rows) and environmental variables (columns) against ovitrap observations of presence and absence.

Fig C – Cross correlation maps for each site (rows) and environmental variables (columns) against ovitrap observations of oviposition abundance.

### Multicollinearity among meteorological variables

In the following figure, we report the Distance correlation coefficient between candidate weather variables for multivariate modeling. For the models, we filtered correlated predictors based on pairwise correlation (r > 0.7), by selecting those predictors with the highest ecological relevance and interpretability.

Fig D – Distance correlation coefficient between candidate weather variables (left : presence model, right ; abundance model)

### Model evaluation

Table E summarizes the predictive performance of the models for each study site. For the machine-learning approach, the ability to discriminate between presence and absence is evaluated using the Area Under the Curve (AUC) of the ROC curve. For the abundance component (restricted to positive observations), model performance is assessed using Spearman correlation and mean absolute error (MAE). Spearman correlation coefficients are accompanied by two-sided p-values from Student’s t-tests. Table E reports the results of paired bootstrap comparisons between the mechanistic and machine-learning models for each site. For each bootstrap iteration, observations were resampled with replacement and correlation coefficients were recomputed for both models using the same samples. The table provides the observed difference in correlation, the mean bootstrap difference, the 95% confidence interval, and a two-sided p-value indicating whether the difference between models is statistically significant. This comparison was performed on the abundance component (positive observations only), where correlation-based evaluation is directly comparable across models.

Table E : Predictive performance of the models for each study site

| **‍Site** | **Mechanistic model** | | **Machine learning model** | | | |
| --- | --- | --- | --- | --- | --- | --- |
|  | **Spearman correlation** | **p-value** | **Spearman correlation** | **p-value** | **MAE** | **‍AUC (presence/absence)** |
| Pérols | 0.7 | 0.00000132 | 0.83 | 5.531362e-11 | 9.63 | ‍0.96 |
| Murviels-les-Montpellier | 0.84 | 0.0000000228 | 0.80 | 2.056340e-08 | 8.45 | ‍1 |
| ‍Bayonne | 0.86 | 0.000000575 | 0.82 | 2.295323e-08 | 6.88 | ‍0.97 |
| ‍Saint-Médard-en-Jalle | 0.9 | 0.000000268 | 0.90 | 4.144167e-07 | 3.95 | ‍0.95 |

Table F– Results of paired bootstrap comparisons between the mechanistic and machine-learning models for each site

| **Site** | **Spearman mechanistic** | **Spearman ML** | **Difference observed** | **Difference bootstrap** | **CI_2.5%** | **CI_97.5%** | **p_value** |
| --- | --- | --- | --- | --- | --- | --- | --- |
| Pérols | 0.7 | 0.83 | 0.12 | 0.12 | 0.01 | 0.29 | 0.0420 |
| Murviels-les-Montpellier | 0.84 | 0.80 | -0.06 | -0.06 | -0.21 | 0.05 | 0.2344 |
| ‍Bayonne | 0.86 | 0.82 | -0.04 | -0.04 | -0.21 | 0.08 | 0.5812 |
| ‍Saint-Médard-en-Jalle | 0.9 | 0.90 | -0.02 | -0.02 | -0.10 | 0.06 | 0.5736 |

### LIME site by site

In the following, we report the detailed LIME (Local Interpretable Model-agnostic Explanations) for each site: Pérols (Fig E), Murviels les Montpellier (Fig F), Bayonne (Fig G), Saint Médard-en-Jalle (Fig H).

Fig E – Local contribution of environmental predictors to predicted mosquito abundance in Pérols using LIME

Fig F – Local contribution of environmental predictors to predicted mosquito abundance in Murviel-les-Montpellier using LIME

Fig G – Local contribution of environmental predictors to predicted mosquito abundance in Bayonne using LIME

Fig H – Local contribution of environmental predictors to predicted mosquito abundance in Saint Médard en Jalle using LIME

Additional consistency Wilcoxon analysis of weather variables and oviposition

Fig I – Consistency analysis per site, year and season between oviposition intensity (boxplots in the first row) against weather variables (boxplots from the second row). For each site and season, the boxplots corresponding to the year of highest oviposition are colored in blue. Then, the boxplots of those combinations of site and season in which the parameters varied consistently(/inconsistently) with oviposition are colored in green(/yellow). Gray boxplots mean lack of significance. The stars indicate the p-value (*: < 0.05, ***: < 0.01, ***: < 0.001) according to the one-to-one Wilcoxon test.

## Additional references

1.	Metelmann S, Caminade C, Jones AE, Medlock JM, Baylis M, Morse AP. The UK’s suitability for *Aedes albopictus* in current and future climates. Journal of the Royal Society Interface. 2019;16(152).

2.	Radici A, Hammami P, Cannet A, L’Ambert G, Lacour G, Fournet F, et al. Aedes albopictus is rapidly invading its climatic niche in France: wider implications for biting nuisance and arbovirus control in Western Europe [Internet]. bioRxiv; 2025 [cited 2025 Feb 22]. p. 2025.02.14.638223. Available from: https://www.biorxiv.org/content/10.1101/2025.02.14.638223v1
