# Supplementary material for: Environmental and demographic determinants of Aedes albopictus seasonal activity in southern France: A modeling study
Source: PLoS Comput Biol. 2026 Sep 10;22(9):e1014799. doi: 10.1371/journal.pcbi.1014799 (PMC13588507; doi:10.1371/journal.pcbi.1014799)
Supplement: Supplementary file 4 [file pcbi.1014799.s005.docx]

Dear Editor,

This letter accompanies the (hopefully last) revision of the manuscript “Environmental and demographic determinants of *Aedes albopictus* seasonal activity in southern France: a modeling study”. Below we provide the response to your comment.

Sincerely,

The authors

## Editor comment

Thank you for the second revision of your manuscript. I have read through the remaining comments and how they were addressed. I think, most of them were addressed satisfyingly. However, I would ask you to revisit one point concerning the interannual Wilcoxon comparisons, which I do not think was adequately resolved.

In response to Reviewer #2's third comment, you added text acknowledging the temporal autocorrelation of the ovitrap observations. However, the added text states that this autocorrelation "leads to an overestimation of the similarity between the seasons" and that significant results are therefore "well-supported." This is the wrong direction: positive autocorrelation among the weekly readings reduces the effective sample size, which inflates the type I error rate --> so you will get more significant (false positive) differences than you would actually have. The passage also wrongly states this as an intra-annual issue, when it is the interannual (2023 vs. 2024) comparisons are actually the ones affected. As written it overstates the robustness of the results. I would recommend revising to something like:

"We compared oviposition abundance, mean temperature and cumulative rainfall between the two years using the Wilcoxon test, applied within each season and site. Because each sample consists of ovitrap readings collected over consecutive weeks, these observations are temporally autocorrelated and do not fully satisfy the test's independence assumption. This tends to inflate the apparent significance of the differences, so these results should be interpreted with caution."

In relation to that point, in the Results section (line 93/94) the sentence “In Pérols, oviposition followed a bimodal pattern in 2023 and was higher in spring and autumn (but not significantly), contrarily to summer ….” I would add a (significant)-bracket after “spring”, because right now it reads like both spring and autumn were non-significant, however only autumn is non-significant. Once this is addressed, I think the manuscript can be accepted for publication.

## Reply

We thank the editor for pointing out our misconception. In fact, we had overlooked the effect that autocorrelation might have on the actual sample size. Furthermore, a early draft of the manuscript included also intra-annual entomological and weather comparisons, subsequently removed for sake of brevity, and this has led us to misinterpret the comment of the reviewer.

We proceeded therefore to modify the paragraph with a slightly revised version of the sentence proposed by the editor, extended to all variables (L. 328-338). Also, we clarified that interannual spring oviposition comparison is significant in Pérols (L. 93-97)
