# Supplementary material for: Environmental and demographic determinants of Aedes albopictus seasonal activity in southern France: A modeling study
Source: PLoS Comput Biol. 2026 Sep 10;22(9):e1014799. doi: 10.1371/journal.pcbi.1014799 (PMC13588507; doi:10.1371/journal.pcbi.1014799)
Supplement: Peer review history

# Peer review: Decision Letter 0

**DOI:** 10.1371/journal.pcbi.1014799.r001

**Authors:** Roger Kouyos, Nanina Anderegg

30 Dec 2025

Temperature is the key weather determinant of Aedes albopictus seasonal activity in southern France

PLOS Computational Biology

Dear Dr. Radici,

Thank you for submitting your manuscript to PLOS Computational Biology. After careful consideration, we feel that it has merit but does not fully meet PLOS Computational Biology's publication criteria as it currently stands. Therefore, we invite you to submit a revised version of the manuscript that addresses the points raised during the review process.

Please submit your revised manuscript by Mar 01 2026 11:59PM. If you will need more time than this to complete your revisions, please reply to this message or contact the journal office at ploscompbiol@plos.org. When you're ready to submit your revision, log on to https://www.editorialmanager.com/pcompbiol/ and select the 'Submissions Needing Revision' folder to locate your manuscript file.

Please include the following items when submitting your revised manuscript:

* A letter that responds to each point raised by the editor and reviewer(s). You should upload this letter as a separate file labeled 'Response to Reviewers'. This file does not need to include responses to formatting updates and technical items listed in the 'Journal Requirements' section below.

* A marked-up copy of your manuscript that highlights changes made to the original version. You should upload this as a separate file labeled 'Revised Manuscript with Track Changes'.

* An unmarked version of your revised paper without tracked changes. You should upload this as a separate file labeled 'Manuscript'.

If you would like to make changes to your financial disclosure, competing interests statement, or data availability statement, please make these updates within the submission form at the time of resubmission. Guidelines for resubmitting your figure files are available below the reviewer comments at the end of this letter

We look forward to receiving your revised manuscript.

Kind regards,

Nanina Anderegg

Guest Editor

PLOS Computational Biology

Roger Kouyos

Section Editor

PLOS Computational Biology

**Additional Editor Comments:**

Overall, the reviewers find the study relevant, however, they raise several points that require revision.

One important concern relates to the interpretation of results, in particular the use of causal language. Reviewers stress that given the limited time span (two years) and number of sites, the analyses support associations and not causal drivers and that this should be acknowledged throughout the manuscript, with non-causal language used accordingly.

Another request by the reviewers is a clearer justification and description of the statistical (ML) modelling choices. This includes the two-stage presence/absence and abundance approach, the selection of predictors and how they were then ultimately combined. For example, it is not clear, why lagged variables were averaged rather than included individually in the RF models, whether alternative modelling strategies were explored (e.g. the use of different lag windows), and how sensitive results are to these choices. Reviewers also note that the inclusion of uncertainty for the RF models would be appreciated. These aspects should be explained more clearly and better justified (or explored, if not yet done).

If these general points, together with the additional comments raised by the reviewers, are adequately addressed, the manuscript can be considered for publication.

**Journal Requirements:**

1) We ask that a manuscript source file is provided at Revision. Please upload your manuscript file as a .doc, .docx, .rtf or .tex. If you are providing a .tex file, please upload it under the item type u2018LaTeX Source Fileu2019 and leave your .pdf version as the item type u2018Manuscriptu2019.

2) Please upload all main figures as separate Figure files in .tif or .eps format. For more information about how to convert and format your figure files please see our guidelines:

https://journals.plos.org/ploscompbiol/s/figures

3) Some material included in your submission may be copyrighted. According to PLOSu2019s copyright policy, authors who use figures or other material (e.g., graphics, clipart, maps) from another author or copyright holder must demonstrate or obtain permission to publish this material under the Creative Commons Attribution 4.0 International (CC BY 4.0) License used by PLOS journals. Please closely review the details of PLOSu2019s copyright requirements here: PLOS Licenses and Copyright. If you need to request permissions from a copyright holder, you may use PLOS's Copyright Content Permission form.

Please respond directly to this email and provide any known details concerning your material's license terms and permissions required for reuse, even if you have not yet obtained copyright permissions or are unsure of your material's copyright compatibility. Once you have responded and addressed all other outstanding technical requirements, you may resubmit your manuscript within Editorial Manager.

Potential Copyright Issues:

- Figure 6. Please (a) provide a direct link to the base layer of the map (i.e., the country or region border shape) and ensure this is also included in the figure legend; and (b) provide a link to the terms of use / license information for the base layer image or shapefile. We cannot publish proprietary or copyrighted maps (e.g. Google Maps, Mapquest) and the terms of use for your map base layer must be compatible with our CC BY 4.0 license.

Note: if you created the map in a software program like R or ArcGIS, please locate and indicate the source of the basemap shapefile onto which data has been plotted.

If your map was obtained from a copyrighted source please amend the figure so that the base map used is from an openly available source. Alternatively, please provide explicit written permission from the copyright holder granting you the right to publish the material under our CC BY 4.0 license.

If you are unsure whether you can use a map or not, please do reach out and we will be able to help you. The following websites are good examples of where you can source open access or public domain maps:

* U.S. Geological Survey (USGS) - All maps are in the public domain. (http://www.usgs.gov)

* PlaniGlobe - All maps are published under a Creative Commons license so please cite u201cPlaniGlobe, http://www.planiglobe.com, CC BY 2.0u201d in the image credit after the caption. (http://www.planiglobe.com/?lang=enl)

* Natural Earth - All maps are public domain. (http://www.naturalearthdata.com/about/terms-of-use/).

4) Please amend your detailed Financial Disclosure statement. This is published with the article. It must therefore be completed in full sentences and contain the exact wording you wish to be published.

- State the initials, alongside each funding source, of each author to receive each grant. For example: "This work was supported by the National Institutes of Health (####### to AM; ###### to CJ) and the National Science Foundation (###### to AM)."

- State what role the funders took in the study. If the funders had no role in your study, please state: "The funders had no role in study design, data collection and analysis, decision to publish, or preparation of the manuscript.".

If you did not receive any funding for this study, please simply state: u201cThe authors received no specific funding for this work.u201d

**Reviewers' comments:**

Reviewer's Responses to Questions

**Comments to the Authors:**

**Please note here if the review is uploaded as an attachment.**

Reviewer #1: Dr. Taconet and colleagues presented an interesting study on Aedes albopictus seasonal activity. Considering the increasing public health significance of Aedes albopictus as a vector of diseases such as dengue and chikungunya which translate to an increased need to control its abundances, the study is undoubtedly interesting and valuable. The study integrates established modeling approaches, mechanistic and random forests (RF) models, to analyze a two-years four-sites ovitrap data in France with corresponding weather time series. The conclusions regarding the extension of the active season due to warmer spring and autumn temperatures are significant for public health, specifically for adjusting arbovirus surveillance calendars.

The primary concern regarding the manuscript is the causal tone adopted by the authors in discussing the observed correlations, which are often presented as determinants or drivers. Despite the use of a mechanistic model, I think that their experimental design, sample size and analysis do not allow for a direct causal interpretation. I suggest authors to tune down the causal language throughout the manuscript.

The modeling efforts are built upon data from only two breeding seasons (and four sites). Temporally, this is a limited database, providing only two independent observations per site for the seasonal occurring events (start, peak, end of seasons). In my opinion, this makes it challenging to pinpoint the exact drivers of mosquito activity. The mosquito species (to the best of my knowledge) is well established in the investigate areas from several years (implying adaptation to local conditions). Therefore, understanding the variations in the start of the season requires data spanning multiple seasons. I would like authors to provide a detailed explanation within the manuscript addressing why the two-season dataset is sufficient to infer key factors, or, alternatively, they must explicitly and clearly outline these temporal limitations in the Discussion section

Another suggestion is that the statistical model section of the Material and methods should be expanded to better describe the type of model used. I assume two random forest models are described here but the terminology Random Forest is not explicitly mentioned.

Some methodological questions/issues follow:

1. The current approach involves two separate models (presence/absence and abundance random forest) separated by an artificial threshold of 0.5 for the presence probability. Did authors consider applying some kind of zero-altered gamma model (or a similar hurdle/two-part model)? This method could predict both presence and abundance without an arbitrary threshold and potentially integrate uncertainty more naturally. Authors should discuss the rationale for preferring the two-separate-model approach and consider also this different statistical approach for comparison.

2. A related issue is how authors dealt with uncertainty. I understand that in the mechanistic model that is absent and probably could be introduced only by considering instead of point-estimate interval distribution for the parameters. Yet uncertainty could be estimated for the RF model. It appears to me that there is no quantification of uncertainty in the random forest prediction, at least as displaied in the figure. There are some papers eg Zhang, et al. "Random Forest Prediction Intervals." The American Statistician,2019. Doi 10.1080/00031305.2019.1585288 dealing with their computation. Was it assessed and/or what was the rationale to prefer point estimate predictions?

3. To the best of my knowledge, random forest models are quite robust to multicollinearity when predicting but can be affected by it when interpreting the importance of each variable. To avoid this, authors selected a set of variables to reduce multicollinearity based on a procedure involving distance and Pearson correlation. First, since their results highlight specific key drivers, I think more details should be given on what variables were highly collinear and were discarded. Second, variable selection can often be performed naturally within the Random Forest framework (as it ranks variables by importance). Was this considered, and why was the external selection procedure preferred/deemed necessary? Third, The rationale for using distance correlation to capture non-linear associations, only to then resort to Pearson correlation (which is less effective at retaining non-linear associations) in a subsequent selection round, is unclear. Please elaborate on this methodological choice. Finally, the caption for the Cross-Correlation Maps needs to clearly explain the meaning of the black and highlighted borders on some squares.

4. Model evaluation: authors used Spearman and Pearson correlation coefficients between predicted and observed egg counts to assess the ability of the two models to capture the temporal dynamic of oviposition. On one hand I agree that if one model prediction correlate with observation it resembles the temporal trend, on the other its absolute values may be critically different, meaning that the model is biologically far off in depicting the population dynamic. That should be mentioned. Moreover, when using correlation, I am not sure what is the impact of having on one hand the values from RF model set to 0 when the presence probability is below 0.5 and in the other the values of mechanistic probably never reaching zero just approximating it. How would the evaluation results change if the RF model output were instead calculated as (predicted abundance) × (probability of presence)? This alternative output should be considered and discussed

5. The values from the mechanistic model are averaged and normalized with respect to their highest value to allow comparison with observed ovitrap data. I acknowledge that explicitly modeling the “capture” process, i.e. modeling the number of eggs that will end up laid in an ovitrap requires unavailable (I think) information (e.g. capture radius and capture rate of ovitraps, contribution of skip oviposition behaviour, …). However, the authors’s modeling choice seems to me to implicitly assume that ovitrap data can accurately represent the population dynamics throughout the season and are not characterized by overdispersion. Two assumptions that are disputed in the literature. Are these assumptions reasonable? Is my reasoning correct? This needs justification or discussion.

6. I assume that in the model beta depends on temperature also for diapausing eggs and there is a missing (T) in the equation.

7. The performance metrics show the RF abundance model generally performs better, with the exception of Bayonne, where the mechanistic model shows a significantly higher correlation. There, the correlation for the RF abundance model in is lower (spearman 0.66, pearson 0.23) compared to the mechanistic model (0.88 and 0.92). This discrepancy for Bayonne is worth exploring and/or discussing in consideration of the author statement regarding RF models globally capturing the seasonal trend.

8. Authors assessed the difference in performance by comparing each model to the data and observing that one correlates better than the others. However, the reader is left wondering if the two performances are statistically different or such differences in the correlation values does not provide enough statistical evidence to reject the hypothesis that both models perform similarly also considering Fig 2.

Reviewer #2: Dear Authors,

I read with interest your manuscript on modelling the oviposition activity of Aedes albopictus in southern France using both a correlative machine learning approach and a mechanistic model. Your work is methodologically robust, the analyses are well executed, and the manuscript is very well written.

However, I would encourage you to reflect on whether the current narrative fully conveys the scientific contribution of the study. While the technical implementation is sound, the novelty and interpretative depth of the findings could be more clearly articulated, particularly with regard to the ecological insights derived from your models.

Let me elaborate:

1. Scope and Novelty

I understand and appreciate that this work is intended as a potential starting point for a machine learning-based forecast system for Ae. albopictus activity in southern France. However, as the manuscript currently stands, the novelty of the scientific contribution is not clear. The central finding, that temperature is the main driver of Ae. albopictus oviposition, is well established in the literature given the ectothermic nature of the species (e.g  https://doi.org/10.3390/insects9040158, https://doi.org/10.1603/0022-2585-38.4.548,  https://doi.org/10.1186/1756-3305-7-338), and is already embedded in the structure of the mechanistic model you used.

2. Lag Structure and Temporal Smoothing of temperature

The incorporation of lagged variables in the correlative model is interesting, and whilst is a solid methodological contribution, I don’t think this is the main novelty of the manuscript. In fact, as you note in the manuscript, this has been explored previously. Additionally, I would question whether such a wide lag window for temperature (0–8 weeks) is biologically meaningful. Does this long averaging period dilute the ecological signal? I understand it is the most important variable in your model, but I wonder if the resulting temperature metric seems heavily smoothed and may obscure critical temporal dynamics, such as cumulative heat effects (e.g. GDD), which have been more widely used in phenology models. Is this smoothed temperature variable still interpretable for early warning purposes?

3. Relative Humidity

I think the role of RH in your model deserves a bit of thinking. Although you rightly mention it as an underappreciated variable (but also this paper that is not currently cited https://doi.org/10.1111/ele.14228) , its temporal and spatial volatility, especially at a weekly scale, raises questions about its reliability in predictive models. Given also your own reservations expressed in L258–261, is it appropriate to retain RH in the final model?

4. Rainfall

I found the precipitation results interesting, particularly the difficulty in establishing a consistent signal, a finding that mirrors the challenges in other studies. This reinforces the complexity of linking rainfall to oviposition at coarse spatio-temporal scales and deserves greater discussion. Additionally we might ask ourself the question if these correlative approaches are the right statistical methods to infer the relationship between rainfall and critical aspects of the species biology. What does the mechanistic model have to say about it? Can you produce a figure similar to Fig.5 but for the effect of precipitation on different life-history trait?.

5. Interpretative Focus

A key unresolved issue is whether the manuscript aims primarily to develop a predictive tool or to gain ecological insight into Ae. albopictus oviposition dynamics. If it is the former, I would expect some form of out-of-sample validation or spatial extrapolation (e.g., using datasets like VectAbundance mentioned in the manuscript). If it is the latter, then results like those in Figures 4 and 5, which potentially reveal biological or climatic thresholds, deserve more analytical space and interpretative emphasis.

I believe this is a high-quality manuscript with the potential to make a meaningful contribution, particularly if the narrative better emphasises either its predictive utility or the biological insights it provides. I hope these comments are helpful in refining the manuscript.

Minor Comments

Introduction

• L63: The phrasing “models assume climate as the main driver” is problematic: mechanistic models are built on this premise, but it’s not an assumption in all contexts. See e.g. Reinhold et al. (2018) for clarification.

• L64: Consider rephrasing “eggs to pupae” as “aquatic stages” to improve clarity.

• L75–78: The mention of arbovirus risk mapping seems out of place in this section, which is otherwise focused on phenology. Consider moving this to the end of the introduction or to the discussion.

Methods

• Have you tested whether an ensemble model (e.g. weighted average of ML and mechanistic models) would improve performance?

• L368: Please define CCM acronym.

• L390: The threshold seems somewhat arbitrary: did you consider optimising it based on model sensitivity or another metric?

Results

• L94–105: Present Figure 1 before Figure 2 for logical consistency.

• Figure 2: In sites sampled every two weeks, how do you determine the true onset/offset of the season? What if the activity peak falls between sampling dates?

• Table 1: Please include a measure of uncertainty or variability (e.g. confidence intervals) for the correlation coefficients.

• Figure 3: Increase label font size for readability.

• L145: Can you estimate and report ath which temperature the inflection point is located?

• Figure 4: The “no activity” symbol might be misinterpreted as indicating vector control, consider a more neutral symbol for winter.

• Consider adding distributions (e.g. quantiles) of the observed response variable by site to contextualise the underestimation of peaks in the correlative model.

Discussion

• L202–204: This sentence is unclear, please revise for clarity.

• L229–230: Again, the shift to arbovirus risk seems abrupt. I would recommend consolidating all disease-related discussion at the end to maintain narrative flow.

• Consider adding a limitation regarding the relatively short time series and the limited number of surveillance sites.

• Ensure species names are italicised consistently in the references.

Reviewer #3: Summary:

Thank you for the opportunity to review this interesting piece of work. It provides an important contribution to the understanding of the ecology and climate suitability of Aedes Albopictus in Southern France. The authors used ovitraps collection data and climatic factors. They analyzed the most contributing weather variables to eggs presence and abundance. To do so, they determined the most important variables and lags through a bivariate correlation test. They used the selected variables and lags to build a machine-learning model (random forest) to predict eggs presence and abundance. They additionally analyzed the interannual variability of eggs counts and their relation with climate-dependent demography parameters from a mathematical model for Ae. Albopictus.

Strengths:

The authors' study covers the important and concerning topic of the spread of the Asian tiger mosquito in Europe. The authors come up with a useful machine-learning based model that deliver impressive predictions, mainly based on temperature. This model can be very useful for a better understanding of mosquito population dynamics, as well as for preparedness against mosquitoes nuisance and infectious diseases outbreaks.

Weaknesses:

Major comments:

1. The selection of lagged weather variables is based on bivariate correlation analyses. This approach is known to be potentially misleading in settings where predictors are highly correlated, as is typically the case for climatic variables. A variable may appear influential at a given lag due to confounding with another correlated variable or lag, rather than reflecting an independent effect. On the contrary, bivariate correlation analyses may fail to identify variables that act primarily at shorter time scales when a strong seasonal signal dominates the response. In such cases, an influential predictor may appear weak or unimportant because its effect is masked by an externally imposed seasonal pattern. This may be the case, for example, for rainfall, which typically exhibits less pronounced annual seasonality than temperature but may still contribute meaningfully to oviposition dynamics at short temporal scales. Consequently, the selected variables and lags may not be identifiable, and their selection should be treated with caution. This limitation should be explicitly acknowledged and discussed.

2. It is unclear why the selected predictors are defined as averages across multiple lag periods, and how this choice follows from the preceding variable and lag selection procedure. This lack of clarity also weakens the interpretation of the statement in the Discussion that "Average temperature of the previous 9 weeks is by far the main determinant of the presence of eggs, and of the previous 5 for their abundance." As the predictors are defined as averages over selected lag windows, it is difficult to disentangle whether these specific lag durations emerge from the data or are, at least in part, imposed by the modeling choices. Clarifying how these lag windows were identified is therefore important for supporting this conclusion.

3. The number of traps in every site influences the egg counts. This is not clear how this aspect is addressed in the methodology.

Minor comments:

1. In the Author summary, the statement that the modelling approaches "qualitatively represent the observed trends" is vague and difficult to evaluate.

2. In the introduction, it is unclear what is actually the research gap, the aim of the paper and how they relate. Stated research gap: "Despite growing research effort to clarify the role of weather determinants of Ae. albopictus activity (15–20), local authorities, public health services and mosquito control agencies often lack of solid scientific evidence to support their policies. These are ultimately jeopardized by climate changes, expected to affect the seasonality of this vector (21)." Do the authors want to provide an early warning system? Or to add on the knowledge of climatic effect on Ae. Albopictus populations? The impact of climate change is not addressed in the paper (but discussed).

3. Throughout the manuscript, the notion of machine learning is associated with statistical model (for instance, "We model oviposition dynamics using both weather-driven (machine learning-based) statistical and mechanistic approaches [...]"). This terminology is potentially misleading, as the methods employed (e.g. random forest–type algorithms) are predictive, data-driven models that do not constitute statistical models in the inferential sense.

4. The structure of the Material and Methods section and the related Results is not straightforward. The manuscript would gain in clarity if the interannual variation topic was entirely grouped after the impact of weather variables.

**********

**Have the authors made all data and (if applicable) computational code underlying the findings in their manuscript fully available?**

The PLOS Data policy requires authors to make all data and code underlying the findings described in their manuscript fully available without restriction, with rare exception (please refer to the Data Availability Statement in the manuscript PDF file). The data and code should be provided as part of the manuscript or its supporting information, or deposited to a public repository. For example, in addition to summary statistics, the data points behind means, medians and variance measures should be available. If there are restrictions on publicly sharing data or code —e.g. participant privacy or use of data from a third party—those must be specified.

Reviewer #1: **No:**  authors stated "All the data and the code will be made available on a Zenodo repository after

acceptance of the article"

Reviewer #2: Yes

Reviewer #3: Yes

**********

PLOS authors have the option to publish the peer review history of their article (what does this mean?). If published, this will include your full peer review and any attached files.

If you choose “no”, your identity will remain anonymous but your review may still be made public.

**Do you want your identity to be public for this peer review?**  For information about this choice, including consent withdrawal, please see our Privacy Policy.

Reviewer #1: No

Reviewer #2: No

Reviewer #3: **Yes:**  Alexis Martin-Makowka

[NOTE: If reviewer comments were submitted as an attachment file, they will be attached to this email and accessible via the submission site. Please log into your account, locate the manuscript record, and check for the action link "View Attachments". If this link does not appear, there are no attachment files.]

**Figure resubmission:**

**Reproducibility:**

?>

# Author comment: Author response to Decision Letter 1

**DOI:** 10.1371/journal.pcbi.1014799.r002

14 Apr 2026

Attachment Submitted filename: ReplyToReviewers.pdf

# Peer review: Decision Letter 1

**DOI:** 10.1371/journal.pcbi.1014799.r003

**Authors:** Roger Kouyos, Nanina Anderegg

13 Jul 2026

PCOMPBIOL-D-25-02163R1

Environmental and demographic determinants of Aedes albopictus seasonal activity in southern France: a modeling study

PLOS Computational Biology

Dear Dr. Radici,

Thank you for submitting your manuscript to PLOS Computational Biology. After careful consideration, we feel that it has merit but does not fully meet PLOS Computational Biology's publication criteria as it currently stands. Therefore, we invite you to submit a revised version of the manuscript that addresses the points raised during the review process.

Please submit your revised manuscript by Sep 12 2026 11:59PM. If you will need more time than this to complete your revisions, please reply to this message or contact the journal office at ploscompbiol@plos.org. When you're ready to submit your revision, log on to https://www.editorialmanager.com/pcompbiol/ and select the 'Submissions Needing Revision' folder to locate your manuscript file.

Please include the following items when submitting your revised manuscript:

* A letter that responds to each point raised by the editor and reviewer(s). You should upload this letter as a separate file labeled 'Response to Reviewers'. This file does not need to include responses to formatting updates and technical items listed in the 'Journal Requirements' section below.

* A marked-up copy of your manuscript that highlights changes made to the original version. You should upload this as a separate file labeled 'Revised Manuscript with Track Changes'.

* An unmarked version of your revised paper without tracked changes. You should upload this as a separate file labeled 'Manuscript'.

If you would like to make changes to your financial disclosure, competing interests statement, or data availability statement, please make these updates within the submission form at the time of resubmission. Guidelines for resubmitting your figure files are available below the reviewer comments at the end of this letter.

As the corresponding author, your ORCID iD is verified in the submission system and will appear in the published article. PLOS supports the use of ORCID, and we encourage all coauthors to register for an ORCID iD and use it as well. Please encourage your coauthors to verify their ORCID iD within the submission system before final acceptance, as unverified ORCID iDs will not appear in the published article. Only the individual author can complete the verification step; PLOS staff cannot verify ORCID iDs on behalf of authors.

We look forward to receiving your revised manuscript.

Kind regards,

Nanina Anderegg

Guest Editor

PLOS Computational Biology

Roger Kouyos

Section Editor

PLOS Computational Biology

**Additional Editor Comments (if provided):**

Dear Andrea Radici

Thank you for the revised version of your manuscript. All three reviewers have reassessed your revised manuscript and were broadly satisfied with the changes. Please address the remaining few minor comments.

**Journal Requirements:**

If the reviewer comments include a recommendation to cite specific previously published works, please review and evaluate these publications to determine whether they are relevant and should be cited. There is no requirement to cite these works unless the editor has indicated otherwise.

1) We have noticed that you have uploaded Supporting Information files, but you have not included a list of legends. Please add a full list of legends for your Supporting Information files after the references list.

2) When completing the data availability statement of the submission form, you indicated that you will make your data available on acceptance. We strongly recommend all authors decide on a data sharing plan before acceptance, as the process can be lengthy and hold up publication timelines. Please note that, though access restrictions are acceptable now, your entire data will need to be made freely accessible if your manuscript is accepted for publication. This policy applies to all data except where public deposition would breach compliance with the protocol approved by your research ethics board. If you are unable to adhere to our open data policy, please kindly revise your statement to explain your reasoning and we will seek the editor's input on an exemption. Please be assured that, once you have provided your new statement, the assessment of your exemption will not hold up the peer review process.

3) We have amended your Competing Interest statement to comply with journal style. We kindly ask that you double check the statement and let us know if anything is incorrect.

**Reviewers' comments:**

Reviewer's Responses to Questions

**Comments to the Authors:**

**Please note here if the review is uploaded as an attachment.**

Reviewer #1: Despite I partially disagree with a couple of responses provided by authors:

1. “This distinction [separating explicitly the ecological processes] was central to our study objectives and is supported by the fact that different predictors and lag structures emerged for each process” zero-altered and hurdle models allows modeling with different covariates the different processes, at least in a Bayesian framework, see Alain Zuur The World of Zero-Inflated Models: Vol 1-3

2. “continuously monitor much larger numbers compared to BG-GAT or BG-Sentinel, whose data are less suited for direct comparison with a mechanistic ODE model” there are various articles using adult data to fit ODE models, see a recente example in Virgillito et al (2025) doi.org/10.1038/s43856-025-00983-8

I think they fully addressed the issues I raised and have no further revision requests

Reviewer #2: Dear Authors,

Thank you for the revised manuscript and for the thorough point-by-point responses to the reviewers' comments. The authors have addressed the concerns raised in the previous round satisfactorily, and I consider the manuscript substantially improved. I am in principle supportive of publication, subject to thee minor points that I’d ask youto address.

1. Season extension claim. The manuscript argues that warmer springs and autumns are likely to extend the active season of Ae. albopictus under climate change. This is a mechanistically coherent argument grounded in the temperature dependence of the species' life-history traits. However, the two-year dataset underlying this study is insufficient to provide empirical support for a directional trend in season length. With only two breeding seasons, any apparent difference in onset or offset dates falls within the range of natural interannual variability and cannot be distinguished from it statistically. The authors should clarify explicitly that the season extension argument is a mechanistic inference rather than an empirical finding, and qualify the relevant statements accordingly.

2. Definition of the ML response variable. The manuscript defines the response variable of the machine learning abundance model as the daily number of eggs per ovitrap (Methods section). This is incorrect. Since ovitraps were sampled every one to two weeks, the recorded egg count represents a cumulative total over the active period of the trap, not a daily value. The response variable should be described as the cumulative number of eggs per trap per sampling period, or equivalently as the mean egg count per sampling interval. The current phrasing risks misleading readers about the temporal resolution and magnitude of the modelled quantity.

3. Wilcoxon test and temporal autocorrelation. The Wilcoxon test is used to compare the distribution of ovitrap records between 2023 and 2024 at each site. Within-season observations at the same site are temporally autocorrelated, which violates the independence assumption of the test and inflates the effective sample size. The authors should acknowledge this limitation in the Discussion, as it further qualifies the strength of the interannual comparisons on which some of the conclusions rest.

Once these aspects are addressed, I think the manuscript can be accepted for publication.

Reviewer #3: I would like to thank the authors for carefully reviewing the manuscript following my comments, and for the clear point-by-point responses. The manuscript has substantially gained in clarity, with the limitations now better stated and the methodology clarified. From my perspective the manuscript is now ready for publication as it is.

**********

**Have the authors made all data and (if applicable) computational code underlying the findings in their manuscript fully available?**

The PLOS Data policy requires authors to make all data and code underlying the findings described in their manuscript fully available without restriction, with rare exception (please refer to the Data Availability Statement in the manuscript PDF file). The data and code should be provided as part of the manuscript or its supporting information, or deposited to a public repository. For example, in addition to summary statistics, the data points behind means, medians and variance measures should be available. If there are restrictions on publicly sharing data or code —e.g. participant privacy or use of data from a third party—those must be specified.

Reviewer #1: None

Reviewer #2: Yes

Reviewer #3: None

**********

PLOS authors have the option to publish the peer review history of their article (what does this mean?). If published, this will include your full peer review and any attached files.

If you choose “no”, your identity will remain anonymous but your review may still be made public.

**Do you want your identity to be public for this peer review?**  For information about this choice, including consent withdrawal, please see our Privacy Policy.

Reviewer #1: No

Reviewer #2: No

Reviewer #3: **Yes:**  Alexis Martin-Makowka

[NOTE: If reviewer comments were submitted as an attachment file, they will be attached to this email and accessible via the submission site. Please log into your account, locate the manuscript record, and check for the action link "View Attachments". If this link does not appear, there are no attachment files.]

**Figure resubmission:**

**Reproducibility:**

To enhance the reproducibility of your results, we recommend that authors of applicable studies deposit laboratory protocols in protocols.io, where a protocol can be assigned its own identifier (DOI) such that it can be cited independently in the future. Additionally, PLOS ONE offers an option to publish peer-reviewed clinical study protocols. Read more information on sharing protocols at https://plos.org/protocols?utm_medium=editorial-email&utm_source=authorletters&utm_campaign=protocols

# Author comment: Author response to Decision Letter 2

**DOI:** 10.1371/journal.pcbi.1014799.r004

31 Jul 2026

Attachment Submitted filename: ReplyToReviewers_2.0.pdf

# Editor report: Decision Letter 2

**DOI:** 10.1371/journal.pcbi.1014799.r005

**Authors:** Roger Kouyos, Nanina Anderegg

12 Aug 2026

PCOMPBIOL-D-25-02163R2

Environmental and demographic determinants of Aedes albopictus seasonal activity in southern France: a modeling study

PLOS Computational Biology

Dear Dr. Radici,

Thank you for submitting your manuscript to PLOS Computational Biology. After careful consideration, we feel that it has merit but does not fully meet PLOS Computational Biology's publication criteria as it currently stands. Therefore, we invite you to submit a revised version of the manuscript that addresses the points raised during the review process.

Please submit your revised manuscript by Oct 12 2026 11:59PM. If you will need more time than this to complete your revisions, please reply to this message or contact the journal office at ploscompbiol@plos.org. When you're ready to submit your revision, log on to https://www.editorialmanager.com/pcompbiol/ and select the 'Submissions Needing Revision' folder to locate your manuscript file.

Please include the following items when submitting your revised manuscript:

* A letter that responds to each point raised by the editor and reviewer(s). You should upload this letter as a separate file labeled 'Response to Reviewers'. This file does not need to include responses to formatting updates and technical items listed in the 'Journal Requirements' section below.

* A marked-up copy of your manuscript that highlights changes made to the original version. You should upload this as a separate file labeled 'Revised Manuscript with Track Changes'.

* An unmarked version of your revised paper without tracked changes. You should upload this as a separate file labeled 'Manuscript'.

If you would like to make changes to your financial disclosure, competing interests statement, or data availability statement, please make these updates within the submission form at the time of resubmission. Guidelines for resubmitting your figure files are available below the reviewer comments at the end of this letter.

As the corresponding author, your ORCID iD is verified in the submission system and will appear in the published article. PLOS supports the use of ORCID, and we encourage all coauthors to register for an ORCID iD and use it as well. Please encourage your coauthors to verify their ORCID iD within the submission system before final acceptance, as unverified ORCID iDs will not appear in the published article. Only the individual author can complete the verification step; PLOS staff cannot verify ORCID iDs on behalf of authors.

We look forward to receiving your revised manuscript.

Kind regards,

Nanina Anderegg

Guest Editor

PLOS Computational Biology

Roger Kouyos

Section Editor

PLOS Computational Biology

**Additional Editor Comments (if provided):**

Thank you for the second revision of your manuscript. I have read through the remaining comments and how they were addressed. I think, most of them were addressed satisfyingly. However, I would ask you to revisit one point concerning the interannual Wilcoxon comparisons, which I do not think was adequately resolved.

In response to Reviewer #2's third comment, you added text acknowledging the temporal autocorrelation of the ovitrap observations. However, the added text states that this autocorrelation "leads to an overestimation of the similarity between the seasons" and that significant results are therefore "well-supported." This is the wrong direction: positive autocorrelation among the weekly readings reduces the effective sample size, which inflates the type I error rate --> so you will get more significant (false positive) differences than you would actually have. The passage also wrongly states this as an intra-annual issue, when it is the interannual (2023 vs. 2024) comparisons are actually the ones affected. As written it overstates the robustness of the results. I would recommend revising to something like:

"We compared oviposition abundance, mean temperature and cumulative rainfall between the two years using the Wilcoxon test, applied within each season and site. Because each sample consists of ovitrap readings collected over consecutive weeks, these observations are temporally autocorrelated and do not fully satisfy the test's independence assumption. This tends to inflate the apparent significance of the differences, so these results should be interpreted with caution."

In relation to that point, in the Results section (line 93/94) the sentence “In Pérols, oviposition followed a bimodal pattern in 2023 and was higher in spring and autumn (but not significantly), contrarily to summer ….” I would add a (significant)-bracket after “spring”, because right now it reads like both spring and autumn were non-significant, however only autumn is non-significant. Once this is addressed, I think the manuscript can be accepted for publication.

**Reviewers' comments:**

[NOTE: If reviewer comments were submitted as an attachment file, they will be attached to this email and accessible via the submission site. Please log into your account, locate the manuscript record, and check for the action link "View Attachments". If this link does not appear, there are no attachment files.]

**Figure resubmission:**

**Reproducibility:**

To enhance the reproducibility of your results, we recommend that authors of applicable studies deposit laboratory protocols in protocols.io, where a protocol can be assigned its own identifier (DOI) such that it can be cited independently in the future. Additionally, PLOS ONE offers an option to publish peer-reviewed clinical study protocols. Read more information on sharing protocols at https://plos.org/protocols?utm_medium=editorial-email&utm_source=authorletters&utm_campaign=protocols

# Author comment: Author response to Decision Letter 3

**DOI:** 10.1371/journal.pcbi.1014799.r006

18 Aug 2026

Attachment Submitted filename: ReplyToReviewers_3.0.docx

# Editor report: Decision Letter 3

**DOI:** 10.1371/journal.pcbi.1014799.r007

**Authors:** Roger Kouyos, Nanina Anderegg

1 Sep 2026

Dear Dr Radici,

We are pleased to inform you that your manuscript 'Environmental and demographic determinants of Aedes albopictus seasonal activity in southern France: a modeling study' has been provisionally accepted for publication in PLOS Computational Biology.

Before your manuscript can be formally accepted you will need to complete some formatting changes, which you will receive in a follow up email. A member of our team will be in touch with a set of requests.

Please note that your manuscript will not be scheduled for publication until you have made the required changes, so a swift response is appreciated.

IMPORTANT: The editorial review process is now complete. PLOS will only permit corrections to spelling, formatting or significant scientific errors from this point onwards. Requests for major changes, or any which affect the scientific understanding of your work, will cause delays to the publication date of your manuscript.

Should you, your institution's press office or the journal office choose to press release your paper, you will automatically be opted out of early publication. We ask that you notify us now if you or your institution is planning to press release the article. All press must be co-ordinated with PLOS.

Thank you again for supporting Open Access publishing; we are looking forward to publishing your work in PLOS Computational Biology.

Best regards,

Nanina Anderegg

Guest Editor

PLOS Computational Biology

Roger Kouyos

Section Editor

PLOS Computational Biology

***********************************************************

# Editor report: Acceptance letter

**DOI:** 10.1371/journal.pcbi.1014799.r008

**Authors:** Roger Kouyos, Nanina Anderegg

PCOMPBIOL-D-25-02163R3

Environmental and demographic determinants of Aedes albopictus seasonal activity in southern France: a modeling study

Dear Dr Radici,

I am pleased to inform you that your manuscript has been formally accepted for publication in PLOS Computational Biology. Your manuscript is now with our production department and you will be notified of the publication date in due course.

The corresponding author will soon be receiving a typeset proof for review, to ensure errors have not been introduced during production. Please review the PDF proof of your manuscript carefully, as this is the last chance to correct any errors. Please note that major changes, or those which affect the scientific understanding of the work, will likely cause delays to the publication date of your manuscript.

Soon after your final files are uploaded, unless you have opted out, the early version of your manuscript will be published online. The date of the early version will be your article's publication date. The final article will be published to the same URL, and all versions of the paper will be accessible to readers.

For Research, Software, and Methods articles, you will receive an invoice from PLOS for your publication fee after your manuscript has reached the completed accept phase. If you receive an email requesting payment before acceptance or for any other service, this may be a phishing scheme. Learn how to identify phishing emails and protect your accounts at https://explore.plos.org/phishing.

Thank you again for supporting PLOS Computational Biology and open-access publishing. We are looking forward to publishing your work!

With kind regards,

Janani Seenivasan

PLOS Computational Biology | Carlyle House, Carlyle Road, Cambridge CB4 3DN | United Kingdom ploscompbiol@plos.org | Phone +44 (0) 1223-442824 | ploscompbiol.org | @PLOSCompBiol
